# Convergence of oncogenic cooperation at single-cell and single-gene levels drives leukemic transformation

**DOI:** 10.1038/s41467-021-26582-4

**Published:** 2021-11-03

**Authors:** Yuxuan Liu, Zhimin Gu, Hui Cao, Pranita Kaphle, Junhua Lyu, Yuannyu Zhang, Wenhuo Hu, Stephen S. Chung, Kathryn E. Dickerson, Jian Xu

**Affiliations:** 1 Children’s Medical Center Research Institute, University of Texas Southwestern Medical Center, Dallas, TX 75390 USA; 2 Department of Pediatrics, Harold C. Simmons Comprehensive Cancer Center, and Hamon Center for Regenerative Science and Medicine, University of Texas Southwestern Medical Center, Dallas, TX 75390 USA; 3 Human Oncology and Pathogenesis Program, Memorial Sloan Kettering Cancer Center, New York, NY 10065 USA; 4 Division of Hematology Oncology, Department of Internal Medicine, and Harold C. Simmons Comprehensive Cancer Center, University of Texas Southwestern Medical Center, Dallas, TX 75390 USA

**Keywords:** Cancer epigenetics, Acute myeloid leukaemia, Tumour heterogeneity, Transcription, Transcriptomics

## Abstract

Cancers develop from the accumulation of somatic mutations, yet it remains unclear how oncogenic lesions cooperate to drive cancer progression. Using a mouse model harboring NRas^G12D^ and EZH2 mutations that recapitulates leukemic progression, we employ single-cell transcriptomic profiling to map cellular composition and gene expression alterations in healthy or diseased bone marrows during leukemogenesis. At cellular level, NRas^G12D^ induces myeloid lineage-biased differentiation and EZH2-deficiency impairs myeloid cell maturation, whereas they cooperate to promote myeloid neoplasms with dysregulated transcriptional programs. At gene level, NRas^G12D^ and EZH2-deficiency independently and synergistically deregulate gene expression. We integrate results from histopathology, leukemia repopulation, and leukemia-initiating cell assays to validate transcriptome-based cellular profiles. We use this resource to relate developmental hierarchies to leukemia phenotypes, evaluate oncogenic cooperation at single-cell and single-gene levels, and identify GEM as a regulator of leukemia-initiating cells. Our studies establish an integrative approach to deconvolute cancer evolution at single-cell resolution in vivo.

## Abstract (web summary)

Identifying how genetic alterations cooperate in cancer is challenging. Here the authors analyze leukemia mouse models with both oncogenic NRAS and EZH2 mutations using single-cell RNA-sequencing, evaluate oncogenic cooperation, and identify GEM as a regulator of leukemia-initiating cells.

## Introduction

Cancers evolve as a consequence of the accumulation of somatically acquired mutations, and their malignant properties reflect the functional cooperation of these mutations^1^. Genetic interactions are central to the selection of variant subclones during cancer evolution, resulting in the acquisition of biological attributes that drive cancer progression and pathogenesis^2^. This is evident in acute myeloid leukemia (AML) and the preleukemic myelodysplastic/myeloproliferative neoplasms (MDS/MPN), a group of genetically and clinically heterogeneous hematological diseases. Recent genomic profiling studies revealed that the vast majority of AML samples harbor approximately a dozen of recurrent genomic alterations with an average of three oncogenic driver lesions per AML genome^3–6^. Although co-occurring somatic mutations are frequently detected in AML patients, it remains elusive how distinct oncogenic drivers cooperate to dysregulate gene expression and cellular differentiation to drive disease progression.

Oncogenic cooperation between different driver mutations has been observed in cell line and mouse models^7^. Specific combinations of AML-associated disease alleles, such as FLT3 and TET2 mutations, confer unique biologic characteristics linked to adverse outcomes^8,9^. However, a major challenge in studying oncogenic cooperation is the lack of high-throughput and high-resolution analysis of molecular changes during the course of cancer progression. Advances in single-cell-based profiling provide opportunities to dissect the molecular processes and cellular state transitions at unprecedented throughput and resolution^10^. While single-cell transcriptomic profiling is widely used to dissect differentiation trajectories in mammalian development, few studies were conducted to interrogate disease progression in vivo using genetically defined model systems^10^.

We previously generated a genetic mouse model with oncogenic mutations in signaling (NRas^G12D^) and epigenetic (EZH2) regulators commonly found in human hematopoietic malignancies^11^. While mice harboring NRas^G12D^ alone developed indolent myeloproliferative neoplasms (MPNs) and EZH2 deletion alone had minimal effects on hematopoiesis, combined NRas^G12D^ and EZH2 mutations cooperatively induced MPN progression to lethal AML^11^. This genetic model permits not only the identification of molecular pathways controlling MPN progression to acute leukemia but also the analysis of the functional cooperation between distinct oncogenic drivers in disease pathophysiology, especially at the single-cell level. Furthermore, the preceding MPN phase in these mice reflects an early stage of cancer development that is inaccessible in most cancer models^12^, and their disease course over an extended period of time provides a unique opportunity for longitudinal studies.

In this work, we employ single-cell transcriptomic profiling to map the cellular states and gene expression alterations of bone marrow (BM) hematopoietic stem/progenitor cells in healthy or diseased mice containing single or combined oncogenic mutations at discrete stages of leukemia progression. We integrate results from histopathology, flow cytometry, leukemia repopulating activity, and leukemia-initiating cell assays to validate the transcriptome-based cellular profiles. We use these approaches to relate developmental hierarchies to leukemia phenotypes, to evaluate functional cooperation between distinct oncogenic drivers at single-cell and single-gene levels, and to identify regulators of leukemia-initiating cells.

## Results

### Single-cell transcriptomic profiling of HSPCs during leukemia progression

**Fig. 1:**
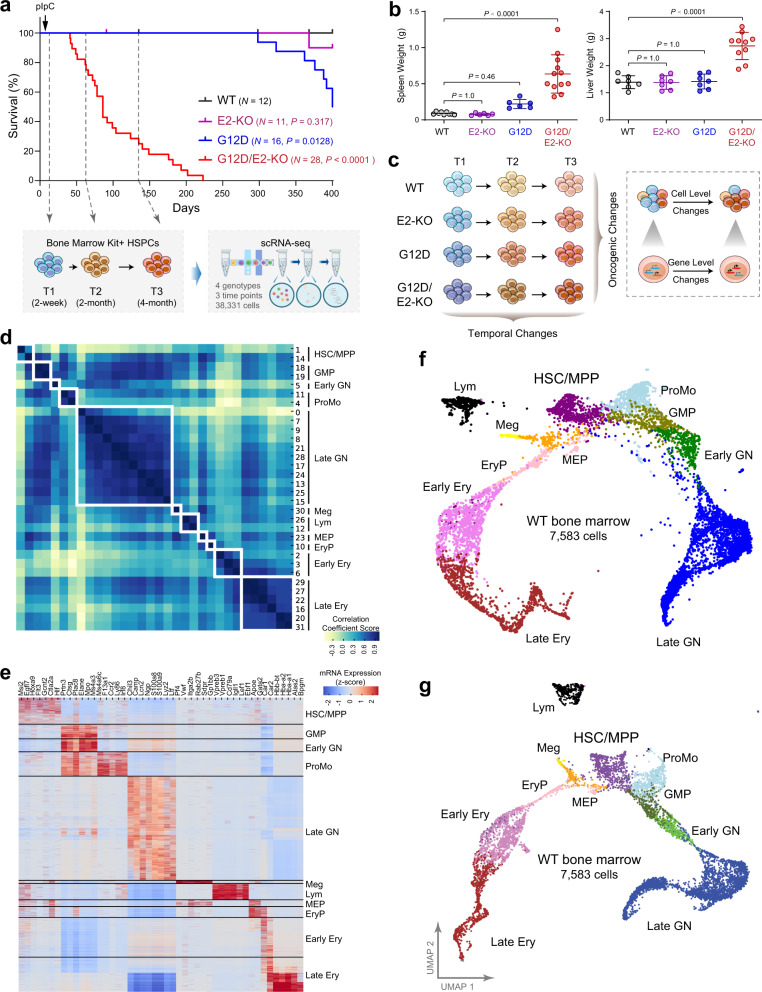
Single-cell transcriptomic profiling of leukemia progression in vivo. **a** Kaplan–Meier survival curves of WT, G12D, E2-KO, G12D/E2-KO mice. *P* values were calculated using the log-rank (Mantel–Cox) test. scRNA-seq was performed on HSPCs of WT, G12D, E2-KO, and G12D/E2-KO mouse bone marrows from distinct stages of leukemia development at disease initiation (2 weeks post-pIpC; T1), evolving MPN (2 months post-pIpC; T2), and blast phase post-MPN leukemia (4 months post-pIpC; T3) as indicated by dash lines. **b** Spleen and liver weight of the indicated genotypes were measured at 10 weeks post-pIpC or when the mice were moribund. Results are mean ± SD (*N* = 9, 6, 6, and 12 for spleen and *N* = 7, 7, 7, and 10 for liver of WT, G12D, E2-KO, and G12D/E2-KO mice, respectively) and analyzed by one-way ANOVA with multiple comparisons. **c** Schematic illustration of the comparative analysis of single-cell transcriptomes between different genotypes (oncogenic changes) or time points (temporal changes). **d** Louvain clustering analysis of single-cell transcriptomes in WT HSPCs. Heatmap is shown for the pairwise Pearson correlations between 32 clusters based on the average expression profiles. Clusters were merged into 11 cell populations based on topology and transcriptional similarity. **e** Expression of the selected cell-type-specific genes in 7583 WT HSPCs ordered by the Louvain-defined clusters. Heatmap shows the normalized gene expression levels. **f** KNN visualization of single-cell transcriptomes in WT HSPCs. Each dot represents a single cell. Cells are colored according to the cell-type annotations. **g** UMAP visualization of single-cell transcriptomes in WT HSPCs. Source data are provided as a Source Data file.

To determine the functional cooperation between oncogenic mutations in signaling and epigenetic molecules in leukemia, we previously generated mice harboring hematopoietic-selective and pIpC-induced (by Mx1-Cre) activation of oncogenic RAS (NRas^G12D^) and inactivation of EZH2, the histone H3-Lys27 methyltransferase^11^ (Fig. 1a). Mice with activation of NRas^G12D+/−^ alone (Mx1-Cre^+^;NRas^G12D+/−^, hereafter G12D) developed chronic myeloproliferation with long latency (median survival >365 days), consistent with an MPN-like phenotype^13^, whereas mice deficient for EZH2 alone (Mx1-Cre^+^;Ezh2^f/f^, hereafter E2-KO) had little effect on hematopoiesis. In contrast, combined NRas^G12D^ activation and EZH2 deficiency (Mx1-Cre^+^;NRas^G12D+/−^;Ezh2^f/f^, hereafter G12D/E2-KO) significantly accentuated disease progression from indolent to lethal MPN and acute leukemia with a shortened survival (median 86 days and mean 103 ± 53 days; *P* < 0.0001 vs. G12D or E2-KO; Fig. 1a). The moribund G12D/E2-KO mice developed severe splenomegaly and hepatomegaly with destructive myelodysplasia not seen in age- and sex-matched wild-type mice (WT; Mx1-Cre^-^) or mice with either mutation alone^11^ (Fig. 1b). We also validated MPN progression to AML by flow cytometry and histopathological analyses in previous studies^11^, thus establishing a genetically defined leukemia model induced by functional cooperation between oncogenic signaling and epigenetic dysregulation.

NRas^G12D^ and EZH2-deficiency-induced leukemia presents important features that recapitulate human AML with preceding preleukemic conditions, including the presence of an indolent MPN phase and the disease course over an extended period of time. We reasoned that the longitudinal analysis of single-cell transcriptomes of evolving hematopoietic cells at different stages of disease progression may allow dissection of the underlying cellular programs and functional cooperation between distinct oncogenic drivers in vivo. To this end, we performed single-cell RNA sequencing (scRNA-seq) analysis of c-Kit^+^ hematopoietic stem/progenitor cells (HSPCs) isolated from WT, G12D, E2-KO, and G12D/E2-KO mouse BMs (Fig. 1c). Since c-Kit is expressed on all hematopoietic stem and early progenitor cells^14^, the use of c-Kit-enriched HSPCs allows an inclusive approach that preserves the relative abundance of progenitor cell states^15^. More importantly, we isolated HSPCs from distinct stages of leukemia development at disease initiation (2 weeks after pIpC-induced NRas^G12D^ activation and/or EZH2 KO; T1, Fig. 1a), evolving MPN (2 months post-pIpC; T2), and blast phase post-MPN leukemia (4 months post-pIpC; T3). Comparing single-cell transcriptomes at discrete time points allowed the analysis of temporal changes during leukemia progression. In addition, comparing cellular and gene-level changes across different genotypes uncovers oncogenic changes caused by either or both oncogenic mutations (Fig. 1c).

We first validated the histopathology of evolving leukemic transformation in G12D/E2-KO mice 4 months post-pIpC. We observed leucoerythroblastic anemia in peripheral blood (PB), increased myeloid:erythroid (M:E) ratio and myelodysplasia in BM, elevated white:red pulp in spleen, and destructive myelodysplasia in spleen and liver (Supplementary Fig. 1a), consistent with previous findings^11^. Moreover, immunohistochemistry analysis of BM and spleen sections revealed the presence of 10–20% or higher c-Kit-positive leukemic blasts, indicating evolving leukemic transformation consistent with post-MPN leukemia (Supplementary Fig. 1a). None of these histological aberrations were observed in pIpC-induced and aged-matched WT, G12D, or E2-KO mice. We next employed a 10× Genomics platform and captured 2519 to 5998 single-cell transcriptomic profiles of BM HSPCs in each genotype at each time point, with a total of 38,331 single cells analyzed at discrete stages of leukemogenesis. After filtering doublets and low-quality cells, we obtained 7583, 7975, 10,611, and 9428 high-quality single-cell transcriptomes from WT, E2-KO, G12D, and G12D/E2-KO for comparative analyses, respectively (Supplementary Data 1).

### Identification of hematopoietic hierarchies in healthy mouse BM

To identify cellular states and gene expression alterations underlying leukemia progression, we developed a custom pipeline to process scRNA-seq data, annotate cell populations, infer developmental hierarchies, and evaluate oncogenic cooperation at single-cell and single-gene levels (Supplementary Fig. 1b). We first identified baseline cellular heterogeneity in BM HSPCs from healthy (WT) mice at time points consistent with evolving leukemia development (T1–T3; Fig. 1a, c). Total 7583 cells from WT HSPCs were grouped into 32-cell clusters using the Louvain algorithm from SCANPY^16^ (Fig. 1d; Supplementary Fig. 1c). We next merged 32 clusters into 11 main cell populations based on the topology and transcriptomic similarity of different clusters (Fig. 1d), and annotated cell populations by the expression of established hematopoietic marker genes^15^ (Fig. 1e; Supplementary Data 2). For instance, the hematopoietic stem cell (HSC)/multipotent progenitor (MPP) population was identified based on the high level expression of a gene signature consisting of *Msi2*, *Flt3*, *Hoxa9*, *Gcnt2*, and *Hlf*, whereas the megakaryocyte-erythroid progenitor (MEP) population was annotated by the expression of both megakaryocytic (*Pf4*, *Vwf*, *Itga2b*, *Rab27b*, *Sdpr*, and *Gp1bb*) and erythroid (*Apoe* and *Gata2*) marker genes. All major hematopoietic cell types were captured in WT HSPCs, including HSC/MPP, MEP, granulocyte–macrophage progenitors (GMP), pro-monocytes (ProMo), lymphocytes (Lym), megakaryocytes (Meg), erythroid progenitors (EryP), early and late erythroid (Ery) cells, and early and late granulocytes (GN) (Fig. 1e; Supplementary Data 2).

To explore the relationships between the identified cell populations, we visualized the captured HSPCs using the *K*-nearest-neighbor (KNN)^17^ and the uniform manifold approximation and projection (UMAP)^18^ approaches, respectively (Fig. 1f, g). Both methods revealed ordered differentiation trajectories starting from HSC/MPP to myeloid, erythroid and lymphoid lineages with a continuum of cells in intermediate states (Fig. 1f, g), consistent with the recent scRNA-seq-based analyses of hematopoietic landscapes^15,19^. Moreover, HSPCs from different time points displayed strong concordance when projected onto the differentiation trajectories, suggesting that the cell state compositions and transitions are largely comparable at these time points in healthy BMs (Supplementary Fig. 1d, e).

### Gene expression changes between and within states in mutant HSPCs

**Fig. 2:**
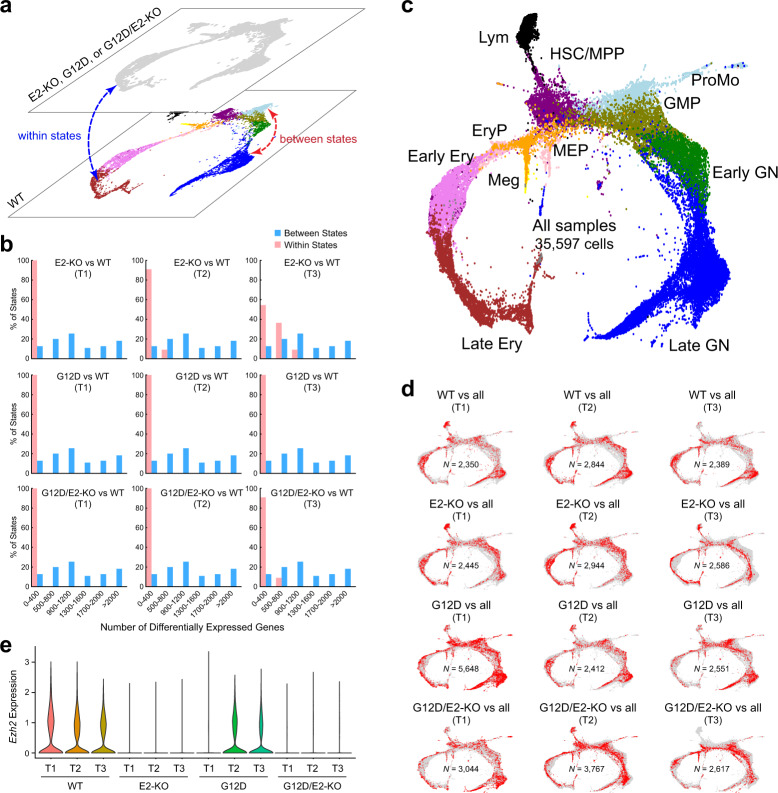
Identification of cellular composition in mutant HSPCs. **a** Annotation of cell populations in mutant HSPCs. The cell state for each cell from E2-KO, G12D, or G12D/E2-KO mutant HSPCs was annotated based on the cell state of their nearest neighbor in WT HSPCs. The differentially expressed genes (DEGs) between WT and mutant HSPCs within the same cell state (within states) or between states within the same genotype (between states) were identified by Wilcoxon rank sum test (adjusted *P* value < 0.01). **b** Number of DEGs within and between cell states. Histograms show the numbers of DEGs between WT and mutant HSPCs within each cell state (*red*) or between different cell states (*blue*). **c** KNN visualization of all single cells by combining 12 samples. Each dot represents a single cell. Cells are colored by cell-type annotations. **d** KNN visualization of single cells from each sample. Each plot shows cells from the indicated genotype and time point (*red*) against all cells from other genotypes and time points (*gray*). **e** Violin plot is shown for *Ezh2* mRNA expression in WT or mutant HSPCs at different time points (T1–T3).

Having established the cell populations and their relationships within differentiation trajectories, we next examined the cellular compositions of HSPCs from E2-KO, G12D, and G12D/E2-KO mutant mice at three time points (T1–T3). We reasoned that the evolving leukemia development may acquire new cellular states or altered differentiation trajectories of the existing cellular states compared to WT HSPCs. To distinguish between these possibilities, we adapted a method with the rationale that the new cellular state would cause widespread gene expression changes in mutant relative to WT HSPCs, and that the differences in gene expression between new and existing cellular states would be comparable to the differences between different existing cellular states^20^ (Fig. 2a).

To this end, we annotated single cells from mutant HSPCs by mapping them to their nearest neighbor cells in WT HSPCs, and identified differentially expressed genes (DEGs) within the same cell states between different genotypes (‘within state’ DEGs in WT vs. E2-KO, G12D or G12D/E2-KO). Compared with DEGs between distinct cell populations in WT HSPCs (‘between state’ DEGs), the numbers of ‘within state’ DEGs among WT and mutant genotypes were significantly less than the numbers of ‘between state’ DEGs in WT HSPCs (Fig. 2a, b; Supplementary Data 3). Using the same criteria across all genotypes and time points, we consistently observed more ‘between state’ DEGs than ‘within state’ DEGs. Of note, though, the numbers of ‘within state’ DEGs increased modestly in E2-KO and G12D/E2-KO HSPCs at late stages of leukemia development (T2 and T3), consistent with a direct role of EZH2 in regulating gene expression during leukemia progression^11,21,22^ (Fig. 2b).

We next combined HSPCs from all genotypes and visualized the differentiation trajectories using KNN-based clustering. Consistent with the results from DEG analysis, we found no new cell clusters with aberrant expression patterns compared to WT HSPCs (Fig. 2c, d). Similar to WT HSPCs (Fig. 1f), KNN analysis of combined HSPCs from 12 samples also occupied a continuum of cellular states with the undifferentiated HSC/MPP population at the core, from which differentiating myeloid, lymphoid, and erythroid lineages appeared (Fig. 2c). Lineage-defining marker genes showed higher expression in terminally differentiated cell states compared to early progenitor cells located closer to the undifferentiated core (Supplementary Fig. 2a). Cell types in mutant HSPCs were annotated according to their nearest neighbor cells in WT HSPCs as described above. When KNN clustering was colored on the basis of different cell types, we observed that mutant cells located in proximity to their WT counterparts, with cells in the same annotated cellular states positioned adjacent to each other (Fig. 2c, d). Finally, we examined the expression of *Ezh2* in single-cell transcriptomes from all samples, and confirmed the significantly lower or absent *Ezh2* expression in the vast majority of E2-KO and G12D/E2-KO HSPCs at three time points (Fig. 2e; Supplementary Fig. 2b).

Together, by single-cell transcriptomic profiling of HSPCs at distinct stages of leukemia development, we found that oncogenic NRas^G12D^ and/or EZH2-deficiency preserved the overall structure of hematopoietic differentiation trajectories similar to HSPCs in healthy BMs; however, more genes displayed altered expression during advanced stages of leukemia progression especially in EZH2-deficient HSPCs.

### NRas^G12D^ and EZH2-deficiency cooperate to dysregulate hematopoietic lineage differentiation

**Fig. 3:**
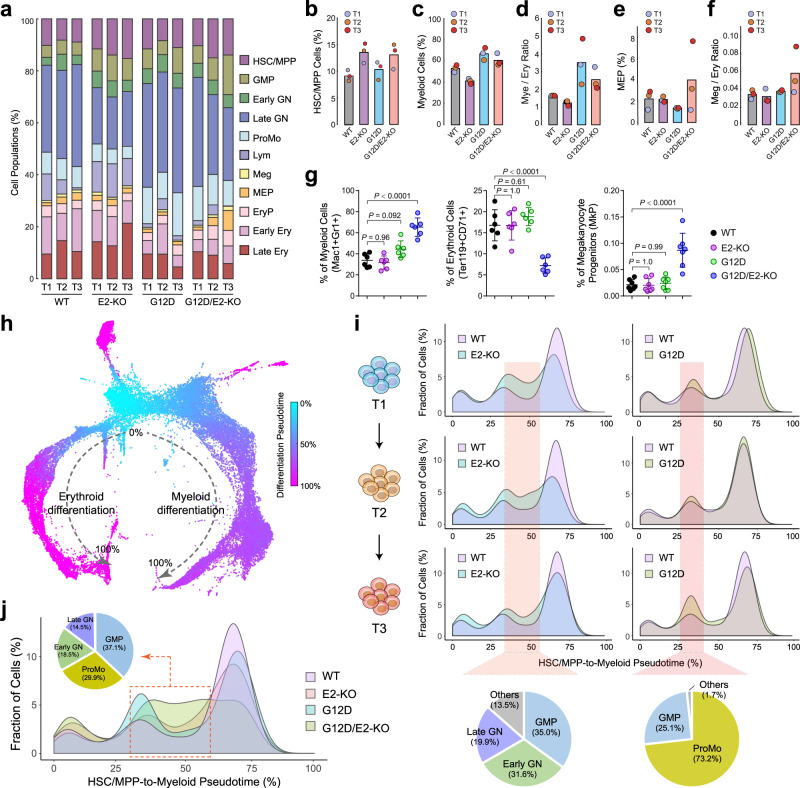
Functional cooperation between NRas^G12D^ and EZH2-deficiency impaired hematopoietic lineage differentiation. **a** The frequencies of annotated cell populations in WT or mutant HSPCs at different time points (T1–T3) are shown. **b** EZH2-deficiency increased the frequency of HSC/MPP population in E2-KO and G12D/E2-KO HSPCs. Bars indicate the mean % of HSC/MPP cells of all time points and colored circles represent individual time points. **c** Expression of NRas^G12D^ increased the frequency of myeloid cells in G12D and G12D/E2-KO HSPCs. **d** Expression of NRas^G12D^ increased the myeloid to erythroid cell ratio in G12D and G12D/E2-KO HSPCs. Bars indicate the mean ratio of myeloid cells of all time points and colored dots represent individual time points. **e** NRas^G12D^ and EZH2-deficiency increased the frequency of MEPs in G12D/E2-KO HSPCs. **f** NRas^G12D^ and EZH2-deficiency increased the megakaryocytic (Meg) to erythroid (Ery) cell ratio in G12D/E2-KO HSPCs. **g** The frequencies of myeloid (Mac1^+^Gr1^+^), erythroid (Ter119^+^CD71^+^) and megakaryocyte progenitor cells (MkP, Lin^−^c-Kit^+^CD150^+^CD41^+^) were determined by flow cytometry in mouse bone marrows at 10 weeks post-pIpC or moribund. Results are mean ± SD (*N* = 6 mice per genotype for myeloid and erythroid cells and *N* = 7 mice per genotype for MkP) and analyzed by one-way ANOVA with multiple comparisons. **h** Differentiation pseudotime of myeloid and erythroid lineages predicted by PAGA. The KNN plot is color-coded by pseudo-temporal order starting from the most immature HSC/MPP cells to mature cells of different lineages. **i** Comparisons of cell density distribution in E2-KO (or G12D) and WT HSPCs along the HSC/MPP-to-myeloid differentiation pseudotime at T1–T3 time points. Pie charts show the cellular composition of the enriched cell populations in E2-KO or G12D HSPCs at T3. **j** The cell density distribution along the HSC/MPP-to-myeloid differentiation pseudotime is shown for the indicated genotypes at T3. Pie chart shows the cellular composition of the enriched cell populations in G12D/E2-KO HSPCs at T3. Source data are provided as a Source Data file.

To investigate the differences in cell state compositions and/or transitions between WT and mutant HSPCs, we calculated the frequency of each cell type in different genotypes (Fig. 3a). Of note, EZH2 loss in E2-KO and G12D/E2-KO HSPCs increased the frequency of HSC/MPP (Fig. 3b). G12D HSPCs showed expanded myeloid lineage cells relative to erythroid and megakaryocytic lineages relative to WT or E2-KO (Fig. 3c, d), consistent with the chronic myeloproliferative phenotypes caused by NRas^G12D^ activation (Fig. 1). The myeloid differentiation bias was also apparent in G12D/E2-KO HSPCs (Fig. 3c, d). These results demonstrate that NRas^G12D^ activation led to skewed hematopoietic differentiation towards myeloid at the expense of erythroid and megakaryocytic lineages. Interestingly, while G12D and E2-KO alone had little effect on the frequency of MEPs which give rise to both megakaryocytes and erythroid cells, G12D/E2-KO HSPCs had significantly and progressively increased frequency of MEPs from T1 to T3 (Fig. 3e). Moreover, the ratio of megakaryocytes to erythroid cells also progressively increased from T1 to T3 during leukemia progression (Fig. 3f).

We validated the single-cell transcriptomic profiles by flow cytometry-based analyses using established lineage markers for myeloid (Gr1^+^Mac1^+^), erythroid (Ter119^+^CD71^+^) and megakaryocytic (Lin^−^c-Kit^+^CD150^+^CD41^+^) cells in mice 4 months post-pIpC (Fig. 3g; Supplementary Fig. 3a). Of note, while NRas^G12D^ activation modestly increased myeloid cells and EZH2-deficiency had no effect, combined NRas^G12D^ and EZH2-deficiency in G12D/E2-KO HSPCs significantly induced the expansion of myeloid lineage cells. Furthermore, while G12D or E2-KO alone had no effect on the frequencies of erythroid cells and megakaryocytes, G12D/E2-KO BM had significantly decreased erythroid cells and increased megakaryocytic progenitors (Fig. 3g). Together with the single-cell analyses, these results illustrate the skewed lineage differentiation of MEPs toward megakaryocytic over erythroid lineages due to the functional cooperation between NRas^G12D^ activation and EZH2-deficiency in HSPCs.

G12D/E2-KO-induced MPNs are characterized by extensive primary myelofibrosis (PMF) in the BM, spleen and liver during advanced stages of disease progression (Supplementary Fig. 3b), however the underlying mechanisms remained unclear^11^. BM fibrosis is the most aggressive form of MPNs associated with more severe diseases, leukemic transformation, and poorer prognosis^13,23,24^. PMF in G12D/E2-KO hematopoietic tissues displayed coarse trichrome-positive collagen fibrosis, dense reticulin fibers with extensive interactions, and osteosclerosis (Supplementary Fig. 3b), consistent with advanced PMF^11,13^. Abnormal megakaryocyte proliferation plays a critical role in the pathogenesis of myelofibrosis^25–27^. Megakaryocytic dysplasia/hyperplasia activates the release of inflammatory cytokines and growth factors such as IFN-γ, CCL5, CXCL5, TGF-β and PDGF that stimulate aberrant proliferation of stromal cells to induce myelofibrosis^25–28^. Therefore, our single cell profiling, flow cytometry, and histopathology uncovered a functional cooperation between NRas^G12D^ activation and EZH2-deficiency to induce MEP expansion and skewed lineage differentiation towards megakaryocytes, leading to the development of advanced myelofibrosis in G12D/E2-KO mice.

### Altered myeloid differentiation by oncogenic cooperation between NRas^G12D^ and EZH2-deficiency

Given the observed alterations in myeloid lineage differentiation, we analyzed cells along the myeloid differentiation path in more detail. Specifically, cells from all samples were combined and colored according to their differentiation pseudotime predicted by partition-based graph abstraction (PAGA)^29^ (Fig. 3h). Comparisons of cell density distributions along the HSC/MPP-to-myeloid differentiation pseudotime showed the accumulation of immature myeloid cells in E2-KO HSPCs at various stages from T1 to T3 (Fig. 3i), consistent with the analysis of cell-type frequencies (Fig. 3a). Of note, G12D HSPCs exhibited a different pattern of cell density distribution with the predominant accumulation of earlier progenitor cells along the myeloid differentiation pseudotime (Fig. 3i). We then analyzed the cellular composition of enriched populations in E2-KO and G12D HSPCs at T3, respectively. The predominantly accumulated cell populations in E2-KO consisted of GMPs (35.0%) and early granulocytes (31.6%), indicating a critical role of EZH2 in the maturation of myeloid lineage cells. By contrast, pro-monocytes (ProMo, 73.2%) and GMPs (25.1%) were the major expanded cell populations in G12D HSPCs (Fig. 3i).

Our results are consistent with previous findings that NRas^G12D^ targets the monocytic lineage cells to induce myelomonocytic proliferation, and that NRAS mutations are frequently identified in patients with chronic myelomonocytic leukemia (CMML) and juvenile myelomonocytic leukemia (JMML)^30–33^. To determine the combined effects of NRas^G12D^ activation and EZH2-deficiency, we analyzed the myeloid differentiation pseudotime in all groups at T3 when post-MPN leukemia was observed in G12D/E2-KO mice. Compared to WT, E2-KO or G12D alone, G12D/E2-KO HSPCs displayed accumulation of both myeloid progenitors (GMP and ProMo) and immature granulocytes and decreased mature myeloid cells (Fig. 3j). Finally, we examined the underlying temporal changes in HSPC composition during leukemia progression by comparing their density distributions along the HSC/MPP-to-myeloid differentiation trajectory at various stages from disease initiation (T1), progression (T2), to blast phase post-MPN leukemia (T3). We observed a gradual accumulation of myeloid progenitors and immature myeloid cells in G12D/E2-KO mice, consistent with the kinetics of evolving MPN progression to leukemic transformation. By contrast, no consistent changes in cell density distribution were observed in WT, E2-KO, and G12D mice (Supplementary Fig. 3c).

Therefore, NRas^G12D^ and EZH2-deficiency have distinct effects on cellular composition along the myeloid differentiation trajectory by expanded myelomonocytic progenitors and impaired myeloid maturation, respectively, and they functionally cooperate to promote the accumulation of both myeloid progenitors and immature myeloid cells in G12D/E2-KO mice.

### Evolving leukemia cells co-express stem cell and myeloid priming genes

**Fig. 4:**
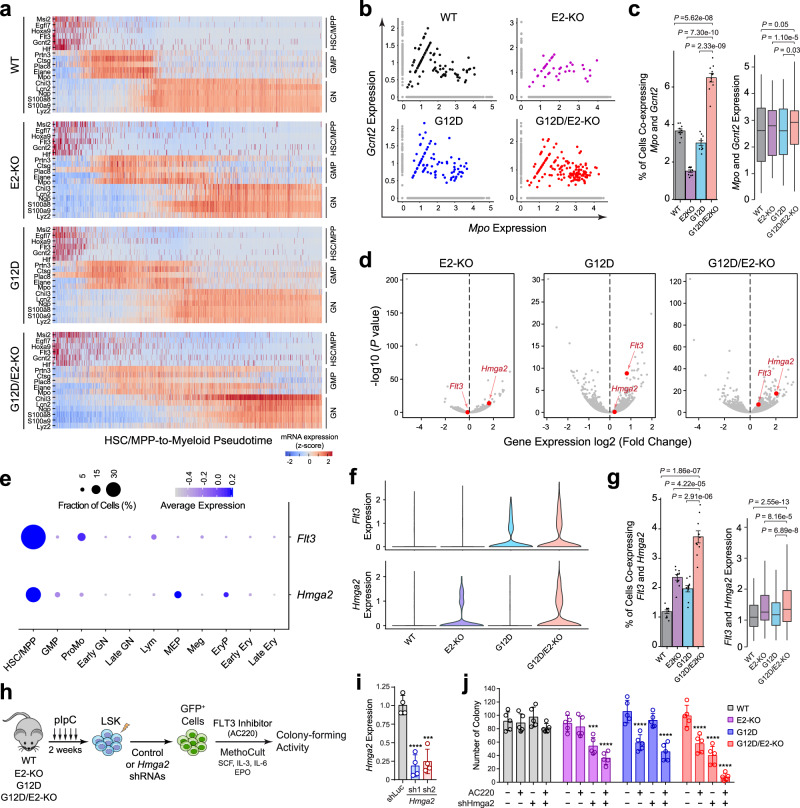
Convergence of dysregulated transcriptional programs in single cells. **a** Expression of marker genes for HSC/MPP, GMP, and granulocytes (GN) along the HSC/MPP-to-myeloid differentiation pseudotime at T3. Heatmap shows normalized gene expression levels. **b** Scatter plots are shown for the expression of *Gcnt2* and *Mpo*. Each dot represents a single cell. **c** Increased frequency of cells co-expressing *Gcnt2* and *Mpo* in G12D/E2-KO HSPCs. Left graph shows the frequency of cells co-expressing *Gcnt2* and *Mpo*. Results are mean ± SD of downsampling iterations (*N* = 1000 cells for 10 iterations). Right graph shows the expression of *Gcnt2* and *Mpo* in cells from different genotypes. Boxes show median of the data and quartiles, and whiskers extend up to 1.5× of the interquartile range. *P* values were calculated by a two-sided *t* test. **d** Volcano plots are shown for bulk RNA-seq. *Hmga2* and *Flt3* were upregulated in E2-KO and G12D HSPCs, respectively. Both *Hmga2* and *Flt3* were upregulated in G12D/E2-KO HSPCs. The *x*-axis shows the log2 fold changes of gene expression and the *y*-axis shows the negative log10 adjusted *P* values. **e** Dot plot shows the expression of *Flt3* and *Hmga2*. The size of the dot indicates the fraction of *Flt3* or *Hmga2*-expressing cells and the color indicates average mRNA levels. **f** Violin plots for *Flt3* and *Hmga2* expression. *Flt3*-expressing HSC/MPP and ProMo cells and *Hmga2*-expressing HSC/MPP and MEP cells were used. **g** Increased frequency of *Flt3* and *Hmga2* co-expressing cells in G12D/E2-KO HSPCs. The graphs were prepared as in panel **c**. **h** Experimental scheme to determine the effect of HMGA2 knockdown and/or FLT3 inhibition on colony-forming ability of HSPCs. **i** Knockdown of HMGA2 was validated by qRT-PCR. Results are mean ± SD (*N* = 4 independent experiments) and analyzed by a one-way ANOVA. ***P < 0.001; ****P < 0.0001. **j** Combined HMGA2 depletion and FLT3 inhibition by AC220 led to more significant defects in colony-formation ability than individual treatments in G12D/E2-KO HSPCs. Results are mean ± SD (*N* = 5 independent experiments) and analyzed by a two-way ANOVA. ***P < 0.001; ****P < 0.0001. Source data are provided as a Source Data file.

Acquiring stem cell gene signatures (or ‘stemness’) is a hallmark of cancer stem cells^34^. In MLL-rearranged AML, leukemia stem cells (LSCs) possessed an immunophenotype and gene expression profile similar to that of normal GMPs but also reactivated a subset of genes highly expressed in normal HSCs. Coexistence of lymphoid-primed multipotent progenitor (LMPP)-like and GMP-like LSCs was observed in other AMLs^35^. To determine whether the evolving leukemia cells within G12D/E2-KO HSPCs acquired features of aberrant stemness, we examined the expression of marker genes for HSC/MPP, GMP, and differentiated granulocytes along the HSC/MPP-to-myeloid differentiation pseudotime. In control mice (WT, E2-KO, and G12D) without leukemia development, single cells were segregated into three distinct groups with a largely exclusive expression of marker genes in HSC/MPP, GMP, and differentiated granulocytes (Fig. 4a). By contrast, HSC/MPP, GMP, and granulocyte marker genes were frequently co-expressed in the same G12D/E2-KO cells, resulting in less distinct segregation of HSC/MPP, GMP, and granulocyte groups along the myeloid differentiation pseudotime (Fig. 4a). We further compared the kinetics of marker gene expression at different time points during leukemia progression. G12D/E2-KO HSPCs displayed progressive changes in marker gene expression resulting in more cells co-expressing HSC/MPP and GMP genes, whereas control HSPCs from WT, E2-KO, or G12D mice showed largely invariant expression patterns with 3 groups segregated across different time points (Supplementary Fig. 4a–d). Further analyses revealed significantly increased frequencies of cells co-expressing HSC/MPP and GMP (or GMP and granulocyte) marker genes (Supplementary Fig. 4e, f), suggesting that oncogenic mutations in NRAS and EZH2 act cooperatively to impair HSC/MPP to myeloid lineage differentiation, resulting in accumulation of cells arrested at development stages co-expressing HSC and GMP (or GMP and granulocyte) genes.

To validate the gene expression patterns at single-gene and single-cell levels, we focused on the HSC/MPP-specific *Gcnt2* gene and the myeloid-specific *Mpo* gene. In WT HSPCs, a small subset (3.6%) of HSPCs co-expressed *Gcnt2* and *Mpo* genes. G12D activation had no effect on *Gcnt2* and *Mpo* co-expression, whereas E2-KO modestly decreased the frequency of *Gcnt2 and Mpo* co-expressing cells. Of note, the frequency of *Gcnt2* and *Mpo* co-expressing HSPCs was significantly increased to 6.4% in G12D/E2-KO mice (Fig. 4b, c). Together, the analyses of global and single-gene expression revealed that the evolving G12D/E2-KO leukemic cells progressively elevate the expression of stemness and myeloid priming genes during leukemia progression.

### Convergence of gene expression alterations by distinct oncogenic drivers

Leukemias evolve from the functional cooperation between driver mutations, which contribute to the biologic and phenotypic properties of the resulting leukemia cells^1,2^. Different oncogenic drivers may cooperate to promote disease progression by acting on the same oncogenic pathways or by the convergent effects on independent pathways perturbed by individual mutations. We previously observed that NRas^G12D^ and EZH2-deficiency converge to reprogram branched-chain amino acid (BCAA) metabolism to drive leukemic transformation by modulating the enzyme and metabolic substrate for BCAA metabolism^11^. Here, we explored whether NRas^G12D^ and EZH2-deficiency may act on independent pathways at the single-cell level to promote leukemia progression.

We first analyzed bulk RNA-seq in HSPCs isolated from control (WT, E2-KO, or G12D) or G12D/E2-KO mice 2 weeks post-pIpC^11^ (Fig. 4d). We found that *Hmga2* was highly upregulated in E2-KO HSPCs, whereas *Flt3* was one of the top upregulated genes in G12D HSPCs. Importantly, both genes were significantly upregulated in G12D/E2-KO relative to WT HSPCs (Fig. 4d), indicating the convergent effects on gene expression due to G12D and E2-KO mutations. *FLT3* is one of the most frequently mutated genes in AML and the majority of FLT3 mutations involve an internal tandem duplication in the juxtamembrane region, resulting in constitutive activation of downstream signaling pathways^36^. Increased expression of *FLT3* is also a risk factor in AML by activating AKT and MAPK pathways, resulting in anti-apoptosis, increased cell survival and abnormal cell proliferation^36^. On the other hand, *HMGA2* is directly regulated by EZH2-catalyzed H3K27me3, whereas elevated *HMGA2* expression is associated with adverse outcomes, serving as a prognostic marker in AML^37,38^. Moreover, HMGA2 plays a crucial role in myeloid differentiation and *HMGA2* silencing induces the differentiation of myeloid leukemia cells^39,40^. Based on these findings, we hypothesized that *Flt3* upregulation by NRas^G12D^ leads to aberrant activation of oncogenic signaling to promote cell proliferation, whereas *Hmga2* activation due to EZH2-deficiency causes myeloid differentiation block, consistent with the altered HSC/MPP-to-myeloid differentiation caused by each mutation (Fig. 3h–j).

To test this, we first determined the cell types that express *Flt3* and *Hmga2* using single-cell transcriptomic profiles. We observed that both genes were predominantly expressed in HSC/MPP cells, with *Flt3* expression also detected in ProMo cells and *Hmga2* detected in MEPs, respectively (Fig. 4e). We next examined *Flt3* and *Hmga2* expression in HSC/MPP, MEP, or ProMo cells, and detected increased numbers of *Hmga2*-expressing cells in E2-KO and *Flt3*-expressing cells in G12D, respectively (Fig. 4f, g), consistent with bulk RNA-seq results (Fig. 4d). More importantly, combined G12D and E2-KO led to a 3.0-fold increase in *Hmga2* and *Flt3* co-expressing cells relative to WT, or 1.6- and 1.8-fold increases relative to E2-KO and G12D alone (Fig. 4g), suggesting that the functional cooperation between EZH2-deficiency and NRas^G12D^ results in the convergence of gene expression alterations at the single-cell level.

Finally, we determined the functional relevance of *Hmga2* and *Flt3* co-expression in G12D/E2-KO HSPCs by measuring the colony-forming activity, which is commonly used to assess hematopoietic or leukemia stem cell activity, with or without FLT3 inhibition and/or HMGA2 depletion (Fig. 4h). We found that depletion of *Hmga2* by two independent shRNAs impaired EZH2-deficient but not G12D HSPCs, resulting in significantly decreased colony-forming activities in E2-KO and G12D/E2-KO HSPCs (Fig. 4i, j). Likewise, FLT3 inhibition by an established FLT3 inhibitor AC220^41^ significantly decreased the colony-forming activities of NRas^G12D^-expressing (G12D and G12D/E2-KO) HSPCs but had little effect on EZH2-deficient cells (Fig. 4j). More importantly, HMGA2 knockdown or FLT3 inhibition alone impaired the clonogenic potential of G12D/E2-KO HSPCs, whereas combining HMGA2 depletion and FLT3 inhibition led to near ablation of the colony-forming activity (Fig. 4j).

Therefore, by integrating single-cell transcriptomics and functional assays, our studies demonstrate that distinct oncogenic drivers promote leukemia progression by activating independent oncogenic pathways in leukemia-initiating cells, and the convergence of different oncogenic pathways in single cells is required for the activity of leukemia-initiating cells.

### Synergistic activation or repression of gene expression by cooperating oncogenic drivers

**Fig. 5:**
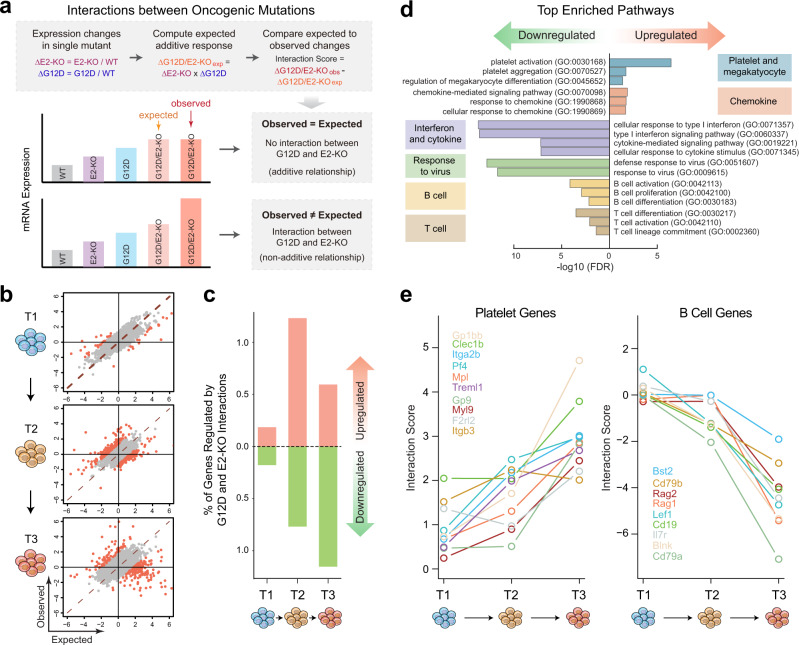
Synergistic regulation of gene expression by cooperating oncogenic drivers in leukemia-initiating cells. **a** Schematic of the analysis of interactions between E2-KO and G12D based on the observed and expected gene expression changes in HSPCs containing single or double mutations. **b** Scatter plots are shown for the observed and expected gene expression changes in G12D/E2-KO HSPCs at three-time points (T1–T3). Each dot represents a single gene. Red dots indicate significantly perturbed genes by the interactions between G12D and E2-KO. **c** The frequencies of genes regulated by G12D and E2-KO interactions are shown at each time point. Upregulated genes were affected by the synergistic interactions of G12D and E2-KO, whereas the downregulated genes were affected by the antagonistic interactions of G12D and E2-KO. **d** The top enriched functional pathways regulated by G12D and E2-KO interactions. The *y*-axis shows the GO terms for the enriched pathways, and the *x*-axis shows the negative log10 (FDR). **e** Interaction scores for platelet (*left*) and B cell genes (*right*) at different time points in G12D/E2-KO HSPCs are shown.

The above analyses illustrate that distinct oncogenic drivers can act on different sets of genes in single cells; however, it remains unknown whether they may also cooperate to dysregulate the same genes additively or synergistically at the single-cell level. To explore this further, we adapted a quantitative metric previously employed to analyze chromatin accessibility^42^ and calculated the interactions between NRas^G12D^ and EZH2-deficiency on target gene expression using the single-cell transcriptomic profiles (Fig. 5a). Specifically, we computed the expression changes for each gene in the single mutant (E2-KO or G12D) relative to WT HSPCs, and the expected additive gene expression changes in G12D/E2-KO HSPCs. We next compared the expected to the observed expression changes in G12D/E2-KO HSPCs and calculated the interaction score, which is defined as the difference between observed and expected gene expression changes (Fig. 5a). Therefore, for any given gene, if the observed expression change in G12D/E2-KO HSPCs matched the expected expression change from the combined effects of each mutation alone, the gene was defined as not being affected by the interactions between NRas^G12D^ and EZH2-deficiency. Conversely, if the observed and expected expression changes significantly differed in G12D/E2-KO HSPCs, the gene was defined to be regulated synergistically or antagonistically due to interactions between NRas^G12D^ and EZH2-deficiency (Fig. 5a).

Using this quantitative metric, we measured the interaction scores for all detected genes using single-cell transcriptomic profiles (Fig. 5b). To compute the permuted background, the observed and expected expression changes for all genes were randomly shuffled, and the differences between all permuted pairs were calculated. We then identified genes significantly affected by interactions between NRas^G12D^ and EZH2-deficiency by comparing the interaction scores with a null distribution generated by permuted gene pairs. Genes with interaction scores beyond 95% of the null values (e.g. top and bottom 5%) were identified to be significantly affected by the antagonistic or synergistic interactions between NRas^G12D^ and EZH2-deficiency (Supplementary Fig. 5a).

By this analysis, we observed that more genes were regulated by progressively increased interactions between NRas^G12D^ and EZH2-deficiency during disease progression from T1 to T3 (Fig. 5b, c). The top enriched cellular pathways for genes upregulated by G12D and E2-KO interactions included platelet development and chemokines (Fig. 5d), consistent with the skewed megakaryocyte differentiation and the development of myelofibrosis in G12D/E2-KO mice (Fig. 3e–g). Conversely, interferon and cytokine, immune response, and B and T lymphoid cell-related pathways were enriched in the down-regulated genes by the interactions between oncogenic mutations (Fig. 5d), consistent with the progressively decreased B and T cell populations in G12D/E2-KO mice (Fig. 3a). Finally, we analyzed the interaction scores for the platelet and B cell signature genes at different stages of disease progression and observed progressively increased interaction scores for the upregulated platelet genes and decreased interaction scores for the downregulated B cell genes (Fig. 5e). By further analyzing cell frequency and gene expression levels in different genotypes, we observed that the upregulation of platelet genes was due to the combined effects on cell frequency and/or gene expression (Supplementary Fig. 5b). Similar results were obtained for the downregulated B cell genes (Supplementary Fig. 5c). These results, together with global gene expression analysis (Fig. 5a–d), illustrate that distinct oncogenic drivers functionally cooperate at single-gene levels to activate or repress gene expression during leukemia progression.

### Identification of candidate regulators of leukemia-initiating cells

AML is characterized by a loose differentiation hierarchy sustained by the self-renewing LSCs or leukemia-initiating cells (LICs) that give rise to a larger population of more mature leukemic blasts^34,43,44^. The presence of LICs contributes to disease prognosis and post-treatment relapse in AML patients^34,44,45^. To determine the regulatory mechanisms by which NRas^G12D^ and EZH2-deficiency cooperate to control aberrant LIC activity, we focused on genes synergistically activated by G12D and E2-KO in HSC/MPP cells, which are known to enrich for LICs^46^.

**Fig. 6:**
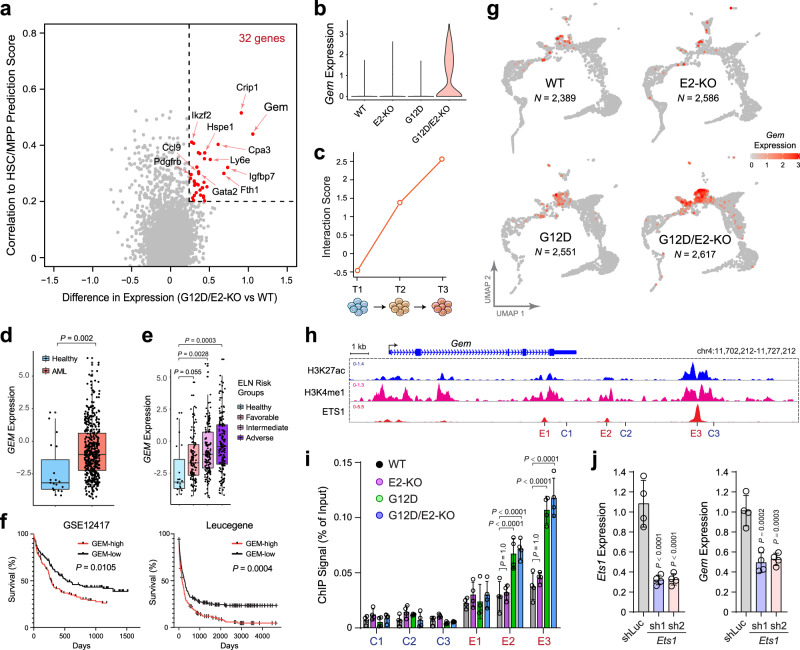
Identification of new candidate regulators of leukemia-initiating cells. **a** Identification of genes highly expressed in HSC/MPP and synergistically activated in G12D/E2-KO HSPCs (*red dots*). Each dot represents a single gene. The *x*-axis depicts the log-transformed expression fold changes between G12D/E-KO and WT HSC/MPP. The *y*-axis depicts the correlation to HSC/MPP prediction scores. **b** Violin plot is shown for *Gem* expression in HSC/MPP from different genotypes. **c** The interaction scores of G12D and E2-KO on *Gem* expression at different time points. **d** *GEM* expression is upregulated in human AML cells relative to control HSPCs in the Beat AML cohorts. Each dot indicates an independent sample (*N* = 19 and 451 for healthy and AML samples). Boxes show the median of the data and quartiles (log2 RPKM), and whiskers extend to 1.5× of the interquartile range. *P* values were calculated by a two-sided *t*-test. **e** Increased *GEM* expression is associated with adverse ELN risk groups in AML. Boxes show the median of the data and quartiles (log2 RPKM), and whiskers extend to 1.5× of the interquartile range. *P* values were calculated by a two-sided *t* test (*N* = 19, 117, 150, and 162 for healthy, favorable, intermediate, and adverse samples, respectively). **f** Increased *GEM* expression associated with poor survival in AML. AML patients were ranked by *GEM* mRNA levels and divided into GEM-low (bottom 50%) and GEM-high (top 50%) groups. *P* values were calculated using the log-rank (Mantel–Cox) test. **g** *Gem* expression is shown on the UMAP graph of the indicated genotypes at T3. Each dot represents a single cell. Color scale shows *Gem* expression level. **h** Browser view of *Gem* locus with H3K27ac, H3K4me1, and ETS1 ChIP-seq in MEL cells. Candidate *Gem* enhancers (E1, E2, and E3) and neighboring control regions (C1–C3) are indicated. **i** ETS1 associates with candidate *Gem* enhancers in HSPCs. Results are mean ± SD and analyzed by two-way ANOVA. **j**, ETS1 depletion impairs *Gem* expression in G12D/E2-KO HSPCs. Results are mean ± SD and analyzed by one-way ANOVA. Source data are provided as a Source Data file.

We first adapted a random forest machine learning method^47^ to identify genes specifically expressed in HSC/MPP cells and dysregulated in G12D/E2-KO HSPCs (Fig. 6a and Supplementary Fig. 6a). We identified 32 HSC/MPP-enriched genes that displayed significant alterations in G12D/E2-KO HSPCs, including a number of known AML-associated genes such as *Gata2*, *Ly6e*, *Igfbp7*, *Crip1,* and *Pdgfrb*^3–6^. More importantly, we also identified several genes whose functional roles in HSCs or LICs are unknown, including *Gem*, a member of the RGK family of GTP-binding proteins within the RAS superfamily^48,49^, and *Cpa3*, a member of the carboxypeptidase A family of zinc metalloproteases^50^. *Gem* is significantly and progressively upregulated in G12D/E2-KO HSPCs due to synergistic activation during leukemia progression (Fig. 6b, c). Expression of *GEM* is also significantly upregulated in human primary CD34^+^ AML cells relative to CD34^+^ HSPCs from healthy donors (Fig. 6d). Increased *GEM* expression is significantly associated with adverse European LeukemiaNet (ELN) risk groups and poorer overall survival in independent AML cohorts^51,52^ (Fig. 6e, f). Moreover, *GEM* expression is increased in AML samples containing PRC2 and/or RAS mutations or higher RAS expression (Supplementary Fig. 6b, c).

Similarly, *Cpa3* is significantly upregulated in G12D/E2-KO HSPCs due to synergistic interactions (Supplementary Fig. 6d, e). *CPA3* expression is also upregulated in human CD34^+^ AML cells relative to control HSPCs and increased *CPA3* correlates with poorer overall survival in AML (Supplementary Fig. 6f, g); however, no significant correlation between *CPA3* expression and ELN risk groups was observed, likely due to its cell-intrinsic and -extrinsic roles as a carboxypeptidase secreted to extracellular space. Out of 32 LIC-associated genes, we noted that higher expression of a number of genes, including *CRIP1*, *FABP5*, *GATA2*, *HSPE1*, *RNABP1*, *GEM* and *CPA3* is significantly associated with poorer survival when analyzed individually or in combination (Supplementary Fig. 6h–o). Together these results uncover a set of candidate regulators of AML-initiating cells.

### GEM is activated by oncogenic cooperation in leukemia-initiating cells

The functional and mechanistic roles of GEM in leukemia development and LIC activity have not been previously investigated. We, therefore, determined how *Gem* is activated by cooperative activation of NRAS and EZH2 mutations. We first observed that NRas^G12D^ activation upregulated *Gem* expression in G12D relative to WT and E2-KO HSPCs, and *Gem* is further upregulated in G12D/E2-KO HSPCs (Fig. 6g and Supplementary Fig. 7a). The RAS/MAPK signaling cascade drives aberrant gene expression through its downstream effector proteins, among which the ETS-family transcription factor ETS1 is particularly important^53^. We hypothesized that NRas^G12D^ may activate *Gem* expression through its downstream ETS-family transcription factors in leukemia-initiating cells. Consistent with this, we identified multiple distal regulatory elements marked by enhancer-associated H3K27ac and H3K4me1 located downstream of *Gem* in multiple murine leukemia cell lines including MEL (E1–E3; Fig. 6h). More importantly, ETS1 strongly associates with these candidate enhancers, in particular E3 located ~17 kb downstream of the *Gem* transcriptional start site (TSS), by ChIP-seq analysis (Fig. 6h). These results suggest that ETS1 may function to activate *Gem* transcription by regulating its distal enhancer elements.

To directly establish the role of the RAS–ETS1 axis in *Gem* regulation, we examined ETS1 chromatin occupancy at the candidate *Gem* enhancers by ChIP experiments in WT, E2-KO, G12D, and G12D/E2-KO HSPCs. Compared to neighboring control genomic regions (C1–C3), the chromatin occupancy of ETS1 is significantly higher at the candidate *Gem* enhancers (Fig. 6h, i). The binding signals at E2 and E3 are further increased in NRas^G12D^-expressing G12D and G12D/E2-KO HSPCs (Fig. 6i). Furthermore, we observed that shRNA-mediated *Ets1* depletion significantly impaired *Gem* expression in G12D/E2-KO HSPCs (Fig. 6j). These results provide direct evidence that NRas^G12D^ activation contributes to *Gem* expression at least in part through its downstream effector ETS1-mediated transcriptional regulation of candidate *Gem* enhancers.

To determine whether and how EZH2/PRC2 regulates *Gem* expression, we first surveyed EZH2-catalyzed H3K27me3 at the *Gem* locus but did not observe any enrichment of H3K27me3 ChIP-seq signals in various hematopoietic and leukemia cell types, suggesting that EZH2 may not directly regulate *Gem* transcription through epigenetic mechanisms. Of note, *Gem* is primarily expressed in HSC/MPP based on our scRNA-seq studies (Fig. 6a, g, and Supplementary Fig. 7a), and EZH2 KO led to the accumulation of HSC/MPP cells in E2-KO and G12D/E2-KO samples (Fig. 3b). These results suggest that EZH2 loss may contribute to *Gem* expression through impaired lineage differentiation, causing accumulation of *Gem*-expressing HSPCs. Moreover, G12D/E2-KO HSPCs showed higher *Gem* expression at the single-cell level compared to G12D HSPCs (Fig. 6b), indicating that there are still yet unknown mechanisms underlying the effect of EZH2 deficiency on *Gem* expression. Taken together, our results support a model whereby NRAS and EZH2 mutations activate *Gem* expression through differential effects on gene transcription and cell differentiation, respectively, and distinct oncogenic mutations may act cooperatively to deregulate gene expression programs in leukemia-initiating cells.

### GEM is a regulator of leukemia-initiating cells

**Fig. 7:**
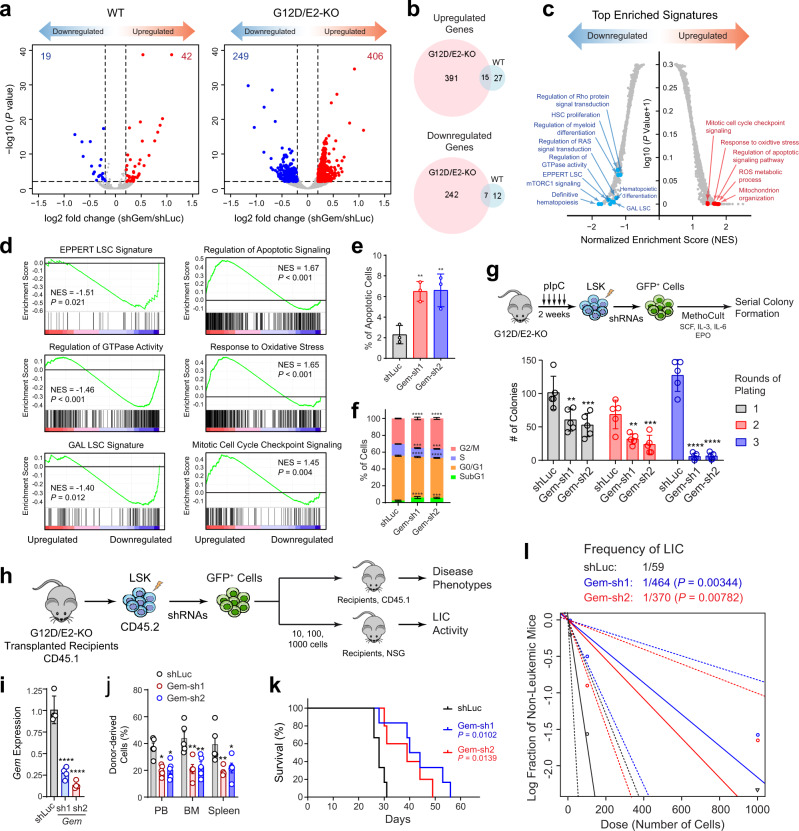
GEM is a candidate regulator of AML-initiating cells. **a** Scatterplots of differentially expressed genes in GEM depleted (shGem) relative to control (shLuc) WT or G12D/E2-KO HSPCs. The numbers of downregulated (*blue* dots) or upregulated (*red* dots) genes are shown. **b** Venn diagrams of differentially expressed genes overlapped between WT and G12D/E2-KO HSPCs upon GEM depletion. **c** Top enriched gene signatures upon GEM depletion in G12D/E2-KO HSPCs. **d** GSEA of top down- or upregulated gene signatures upon GEM depletion in G12D/E2-KO HSPCs. **e** GEM depletion increased apoptosis (Annexin V^+^) in G12D/E2-KO LSK cells. Results are mean ± SD (*N* = 3 experiments) and analyzed by one-way ANOVA. **P < 0.001. **f** GEM depletion led to cell cycle arrest in G12D/E2-KO LSK cells. Results are mean ± SD (*N* = 3 experiments) and analyzed by two-way ANOVA. **P < 0.001; ***P < 0.0001. **g** Experimental scheme to determine the effect of GEM knockdown on G12D/E2-KO HSPCs (*top*). Colony formation assays of cells transduced with the indicated shRNAs are shown on the bottom. Results are mean ± SD and analyzed by a two-way ANOVA. **P < 0.01; ***P < 0.001; ****P < 0.0001. **h** Experimental scheme to determine the effect of GEM depletion on leukemia-initiation cells. **i** Validation of GEM knockdown in G12D/E2-KO HSPCs by qRT-PCR. Results are mean ± SD and analyzed by a one-way ANOVA. ****P < 0.0001. **j** Frequencies of donor-derived (CD45.2^+^) cells in PB, BM, and spleen in mice transplanted with G12D/E2-KO cells with control (shLuc) or GEM-targeting shRNAs at 28 days post-transplantation. Results are mean ± SEM and analyzed by a two-way ANOVA. *P < 0.05; **P < 0.01. **k** Survival curves of mice transplanted with G12D/E2-KO cells with control (shLuc) or GEM-targeting shRNAs. *P* values were calculated using the log-rank (Mantel–Cox) test. **l** LIC frequencies were determined by limiting dilution at three doses (10, 100, and 1000 cells) and transplantation to NSG mice (*N* = 5 per group). G12D/E2-KO HSPCs were transduced with control (shLuc) or GEM-targeting shRNAs. *P* values were calculated by chi-squared test. Source data are provided as a Source Data file.

To establish the functional role of the identified candidate regulators of LICs based on single-cell analysis, we focused on GEM for detailed studies. We first performed an RNA-seq analysis of WT and G12D/E2-KO HSPCs containing control (shLuc) or GEM depletion (shGem) to determine the cellular pathways regulated by GEM. We found that GEM depletion in WT HSPCs leads to only modest changes in gene expression (19 downstream and 42 upregulated genes; FDR-adjusted *P* ≤ 0.01 and log2 fold-change ≥0.2) but significant gene expression changes in G12D/E2-KO LICs (249 and 406 down- and upregulated genes; Fig. 7a, b; Supplementary Data 4). GEM depletion in G12D/E2-KO LICs significantly increased the expression of gene signatures associated with apoptotic signaling, response to oxidative stress, mitochondrial organization, and cell cycle checkpoint. By contrast, gene signatures associated with Ras, Rho, and small GTPase-mediated signal transduction, HSC proliferation, and leukemia stem cells (LSC) ^54,55^ are significantly downregulated (Fig. 7c, d). Consistent with the RNA-seq results, we observed that GEM depletion in G12D/E2-KO LICs significantly increased cell apoptosis and cell cycle arrest (Fig. 7e, f), illustrating a critical role for GEM in the proper control of cell proliferation, cell cycle progression, and apoptosis signaling in LICs. By contrast, depletion of GEM or its regulator ETS1 had little effect on apoptosis or cell cycle of WT HSPCs (Supplementary Fig. 7b–d), consistent with minimal gene expression changes upon GEM depletion in WT HSPCs (Fig. 7a, b). Of note, hematopoietic-selective *Ets1* knockout (by Vav-Cre) in mice had no effect on normal HSPCs except the impaired T cell development^56^, suggesting that ETS1 and GEM are selectively required for the propagation of G12D/E2-KO LICs.

To directly examine the functional roles of GEM in LICs, we performed a series of ex vivo and in vivo studies. We first measured the colony-forming activity of G12D/E2-KO HSPCs upon serial replating (Fig. 7g). While control HSPCs (shLuc) showed efficient colony-forming activity during three rounds of plating, GEM-depleted HSPCs (Gem-sh1 and Gem-sh2) were significantly impaired in clonogenic activity, resulting in progressive loss of the colony-forming ability (Fig. 7g). We next determined the requirement of GEM for leukemia development in vivo by transplantation of control (shLuc) or GEM-depleted G12D/E2-KO HSPCs (CD45.2) to congenic recipient (CD45.1) mice (Fig. 7h). Notably, GEM depletion ameliorated G12D/E2-KO-induced leukemic phenotypes and delayed disease progression in transplanted recipient mice, resulting in significantly reduced leukemic burden in hematopoietic tissues (PB, BM and spleen) and prolonged survival (Fig. 7i–k). Finally, we measured LIC cell activity by limiting dilution and transplantation assays (Fig. 7h). GEM depletion significantly impaired LIC activity of G12D/E2-KO HSPCs, resulting in 6.3- to 7.9-fold decreases in LIC frequency relative to control (shLuc) HSPCs (*P* = 0.00782 and 0.00344, respectively; Fig. 7l).

Given that mutations of epigenetic regulators (e.g. EZH2) often co-occur with mutations in signaling (e.g. NRAS) or lineage-regulating transcriptional factors to drive clonal progression of myeloid malignancies^57^, our results suggest that the aberrant upregulation of GEM may be generally observed in AML through combinations of oncogenic mutations. Consistent with this notion, *GEM* expression is also increased in AML cases containing other genetic lesions, such as TET2 and ASXL1 mutations, in the ECOG 1900 cohorts^3,58^. More importantly, *GEM* expression is significantly increased in high risk lesions such as monosomy 7, EVI1-positive, del(5q), and complex karyotype (Supplementary Fig. 7e). These findings establish GEM as not only a regulator of AML-initiating cells, but also a potential prognosis biomarker for stratification of AML risk groups and/or clinical management.

Taken together, by integrating single-cell transcriptomics, leukemia phenotypes and repopulating activity, and leukemia-initiating cell assays, we uncovered functional cooperation between distinct oncogenic drivers at single-cell and single-gene levels. Our findings support a model whereby cooperating oncogenic mutations act on the same and distinct pathways to control progressive alterations of cellular differentiation, whereas the convergence of oncogenic cooperation at single-cell and single-gene levels rewires gene programs to promote leukemia-initiating cell activity and cancer progression.

## Discussion

A long-standing question in cancer biology is how distinct oncogenic mutations cooperate to promote cancer pathogenesis and malignant properties. Oncogenic cooperation has been studied historically using cell line or animal models^7,59^. While cell lines provide convenient tools for manipulating tumor cells in vitro, they lack the contextual interactions required for cancer development in their in vivo microenvironment. On the other hand, genetically engineered mouse models provide opportunities to examine the functional effects of predefined oncogenic mutations in relevant cell types in vivo. Moreover, bulk tumor-based analyses can be limiting for understanding molecular alterations caused by oncogenic interactions within and between cell types or single cells. As such, a major challenge has been the lack of high-throughput, high-resolution, and longitudinal analysis of oncogenic cooperation during cancer progression.

In this study, we leveraged a genetic mouse model recapitulating leukemia progression driven by oncogenic cooperation between signaling (NRas^G12D^) and epigenetic (EZH2) alterations, and performed time-resolved single-cell transcriptomic profiling to interrogate the molecular and cellular changes during leukemia development. We compared BM HSPCs in healthy and diseased mice containing single or combined oncogenic mutations at distinct stages of leukemia development from disease initiation, evolving MPN, to post-MPN leukemia. Comparing single-cell transcriptomes across different genotypes with or without leukemia development provides insights into dysregulated cellular differentiation and gene programs responsible for the malignant phenotypes. In addition, the analysis of time-resolved single-cell gene expression kinetics provides an unprecedented view of cellular state transitions and altered differentiation trajectories during the course of leukemia progression.

Co-occurring mutations in epigenetic regulators and signaling factors are common in human myeloid malignancies^57^. Our focused studies on EZH2 and NRAS mutations provide an opportunity to dissect how distinct oncogenic pathways converge at the single-cell and single-gene levels to cause aberrant gene expression and leukemia progression. Specifically, at the cellular level, we found that NRas^G12D^ led to accumulation of myeloid progenitors and skewed hematopoietic differentiation towards myeloid lineage at the expense of erythroid/megakaryocytic lineages, whereas EZH2-deficiency impaired myeloid cell maturation along the HSC to myeloid differentiation axis resulting in accumulation of immature myeloid cell populations. These results revealed distinct mechanisms by which individual oncogenic drivers impact hematopoietic lineage differentiation at single-cell levels. More importantly, combined NRas^G12D^ and EZH2-deficiency significantly and progressively enhanced the expansion of myeloid cell compartments, resulting in accumulation of myeloid progenitors, immature myeloid cells and megakaryocytes, but decreased lymphoid and erythroid cells, illustrating that the oncogenic cooperation between NRas^G12D^ and EZH2-deficiency manifested a more profound impact on hematopoietic differentiation to drive leukemia progression.

At the gene level, we showed that NRas^G12D^ and EZH2-deficiency individually regulated a set of independent gene targets such as *Flt3* and *Hmga2*; however, combined NRas^G12D^ and EZH2-deficiency resulted in the convergence of dysregulated transcriptional profiles in the same cells that contributed to the malignant properties of HSPCs in double mutant mice. More importantly, we provided evidence that NRas^G12D^ and EZH2-deficiency also acted synergistically to control the expression of the same genes, such as genes required for platelet and B cells, by acting on gene transcription and/or cell differentiation. These synergistically dysregulated transcriptional profiles were superimposed on mutation-specific alterations in malignant progenitors, resulting in conflated stemness and myeloid gene expression programs. The identification of gene signatures synergistically regulated by NRas^G12D^ and EZH2-deficiency also provided a mechanistic explanation for leukemia-associated histopathological features, including abnormal megakaryocyte proliferation, primary myelofibrosis, and impaired lymphoid development, in the combined but not single mutant animals. Hence, these findings provide insights into the fundamental question of how cooperating mutations in epigenetic and signaling factors may be positively selected during clonal evolution to drive leukemic transformation. Our studies also emphasize the importance of cross comparisons at single-cell levels between different genotypes or time points for the identification of biologically relevant molecular changes in cancer progression.

AML contains a loose differentiation hierarchy consisting of leukemia-initiating cells (LICs) and more mature leukemic blasts^34,43,44^. LICs are capable of repopulating the disease and have been implicated in post-treatment relapse in leukemia patients^34,44,45^. We reasoned that correlating single-cell transcriptome-based developmental hierarchies with leukemia phenotypes may identify the underlying gene programs responsible for the aberrant LIC activity. Through the random forest-based machine learning approach^47^ we identified a set of candidate genes such as *Gem* that are highly expressed in HSC/MPP but dysregulated due to the oncogenic cooperation between NRas^G12D^ and EZH2-deficiency. Furthermore, using serial replating colony-formation, leukemia repopulation, and LIC limiting dilution assays, we established the functional roles for GEM as a regulator of LIC activity. Aberrant activation of *GEM* was also observed in human AML associated with a spectrum of genetic lesions and correlated with poor prognosis. Given that *GEM* expression is highly enriched in normal HSC/MPP and significantly upregulated in LICs, the observed correlation between *GEM* expression and AML phenotypes may reflect an expansion of *GEM*-expressing hematopoietic stem and/or progenitor cells in high-risk leukemia. These results demonstrate that the integrative analysis of single-cell transcriptomic profiles, annotation of cell populations and development trajectories, leukemia phenotypes, and functional studies can reveal molecular targets and cellular pathways required for cancer-initiating cell function. Despite these advances, it is important to note that the current study does not provide information about the combinatorial effects caused by sequential acquisition of mutations during disease progression, and more advanced mouse modeling that recapitulates physiological disease trajectories would be necessary in future studies.

In conclusion, we leveraged the genetically engineered cancer models, high-resolution single-cell transcriptomics, histopathology, and functional studies to investigate the cellular and molecular alterations caused by oncogenic interactions during cancer progression. Our findings reveal the altered cellular composition and developmental hierarchies corresponding to leukemia phenotypes, uncover the aberrant transcriptional programs in evolving leukemia cells, and identify regulators of leukemia-initiating cells. These results support a model that distinct oncogenic mutations cooperate at single-cell and single-gene levels, and the convergence of dysregulated gene programs rewires differentiation trajectories to promote leukemia progression. Hence, our studies not only uncover cellular and gene programs controlling leukemic transformation, but also provide an integrative approach for in vivo analysis of cancer evolution.

## Methods

### Mice

Ezh2 floxed mice and NRas^G12D^ mice containing the LSL-NRas^G12D+/−^ knock-in allele were generated as previously described^11,60^. Mx1-Cre mice were obtained from the Jackson Laboratory. All mouse lines were maintained on a C57BL/6 background. All mice were housed under a 12-h light–dark cycle, 75 °F, and 35% humidity in the Animal Resource Center at the University of Texas Southwestern Medical Center (UTSW). All mouse experiments were performed under protocols approved by the Institutional Animal Care and Use Committee of UTSW.

### RNA isolation and qRT-PCR analysis

RNA was isolated using Qiagen RNeasy Mini or Micro Plus kit and reverse-transcribed using iScript cDNA Synthesis Kit (Bio-Rad) following manufacturers’ protocols. Quantitative RT-PCR was performed on CFX384 Touch Real-Time PCR Detection System (Bio-Rad). PCR was performed in triplicates with the iQ SYBR Green Supermix (Bio-Rad) with the following parameter: 95 °C (3 min), 45 cycles of 95 °C (15 s), 60 °C (30 s), and 72 °C (30 s). Primer sequences are listed in Supplementary Data 5.

### Flow cytometry and MACS cell separation

Flow cytometry analyses of BM cells were performed as described previously with modifications^11,61^. Briefly, BM cells were obtained by crushing femurs, tibias, vertebrae and pelvic bones with a mortar in Ca^2+^ and Mg^2+^-free Hank’s buffered salt solution (HBSS; Gibco) supplemented with 2% heat-inactivated bovine serum (HIBS, Gibco). All cell suspensions were filtered through 70 μm cell strainers and cell numbers were determined using a Vi-CELL cell viability analyzer (Beckman Coulter). To enrich c-Kit^+^ cells, BM cells were stained with c-Kit-APC780 followed by anti-APC-conjugated microbeads. Cells were then separated by LS columns (Miltenyi Biotec) at 4 °C. CD2, CD3, CD5, CD8, B220, Gr1 and Ter119 antibodies were used to exclude lineage-positive cells. DAPI (4,6-diamidino-2-phenylindole) or PI (propidium iodide) was used to distinguish live or dead cells in flow cytometry assays. FACSAria or FACSCanto flow cytometer (BD Biosciences) was used for flow analysis. Cell sorting data were acquired and analyzed by FACSDiva (BD Biosciences). All antibodies used for cell isolation and flow cytometry are listed in Supplementary Data 6.

### scRNA-seq Library preparation and sequencing

G12D/E2-KO mice had disease progression from indolent to lethal MPN and acute leukemia with a shortened survival (median 86 days and mean 103 ± 53 days; range 41–223 days). Given that we administered pIpC to mice at around 6-week old, the post-pIpC survival was median 44 and mean 61 days. We, therefore, chose 2 weeks (T1), 2 months (T2), and 4 months (T3) post-pIpC-induced NRas^G12D^ activation and EZH2 inactivation to represent disease initiation (T1), MPN progression (T2), and leukemia transformation (T3). BM c-Kit^+^ enriched cells from WT, E2-KO, G12D, and G12D/E2-KO mice at each time point were immediately processed for library preparation using the 10x Genomics Chromium Single Cell 3′ Reagent Kit following manufacturer’s protocols. Libraries were quantified using the double-stranded DNA High-Sensitivity Assay Kit (Invitrogen) on the Qubit fluorometer and the Agilent 2200 TapeStation systems. Indexed libraries were pooled and sequenced on an Illumina NextSeq 500 system following the recommended sequencing parameters from 10x Genomics.

### scRNA-seq data processing and filtering

Raw sequencing reads were first pre-processed with 10× Genomics Cell Ranger v2.0.2 pipeline and aligned to the mouse mm10 reference genome. Each sample was processed separately. Putatively stressed or dying cells with >10% of their transcripts coming from mitochondrial genes were excluded for analysis. Putative doublets were removed by Scrublet^62^. After cell filtering, we detected the following numbers of cells, respectively: WT T1, 2350; G12D T1, 5648; E2-KO T1, 2445; G12D/E2-KO T1, 3044; WT T2, 2844; G12D T2, 2412; E2-KO T2, 2944; G12D/E2-KO T2, 3767; WT T3, 2389; G12D T3, 2551; E2-KO T3, 2586; G12D/E2-KO T3, 2617. The detailed quantitative analysis of scRNA-seq datasets is shown in Supplementary Data 1.

### Clustering and annotation of cell populations in WT HSPCs

7583 WT HSPCs from T1–T3 time points were clustered into 32 cell clusters using the Louvain algorithm from SCANPY^16^. Louvain is a hierarchical clustering algorithm that recursively merges communities into a single node and executes the modularity clustering on the condensed graphs (https://neo4j.com/docs/graph-algorithms/current/algorithms/louvain/; https://github.com/vtraag/louvain-igraph; 10.5281/zenodo.595481). The topology of different clusters was visualized by PAGA, which provides an interpretable graph-like map of the arising data manifold based on estimating connectivity of manifold partitions and preserving the global topology of data^29^. Then 32 cell clusters were further merged into 11 cell populations based on similarities in transcriptomes and the topology of cell clusters.

### KNN and UMAP visualization

Two dimensionality reduction methods, KNN^17^ and UMAP^18^, were used to visualize single-cell RNA-seq data. SPRING^63^ was employed to generate KNN graph which connects each cell to its five nearest-neighbor cells based on their transcriptomic similarity. KNN graph was visualized in two-dimensional space by SPRING using a force-directed layout. For UMAP visualization, we used Seurat v3.2.0 implementation in R with default parameters^64^.

### Annotation of cell populations in mutant HSPCs

Cells from mutant HSPCs (E2-KO, G12D, and G12D/E2-KO) were projected into the same principal component space as the WT HSPCs and mapped to their most similar WT neighbors as previously described^15^. Briefly, gene counts in each cell were normalized by the total counts. Highly variable genes were *z*-score normalized and used to identify the top 50 principal components in WT HSPCs. Then mutant HSPCs were *z*-score normalized and transformed into the WT principal component space. Lastly, each mutant cell was mapped to its closest WT neighbor in principal component space (Euclidean distance). We tried an alternative approach by the integration and label transfer function implemented in Seurat^64^, which also projects the PCA structure of a reference onto the query to predict the cell types of queried mutant cells, and got similar results. DEGs between different cell states in the same genotype or within the same cell state in different genotypes were identified by Wilcoxon rank-sum test implemented by Seurat with adjusted *P* value < 0.01.

### Analysis of single-cell differentiation trajectories

We used PAGA in SCANPY v1.4 to infer the differentiation pseudotime^29^. The graph abstraction algorithm reconciles clustering and trajectory inference by explaining data variability in terms of both discrete and continuous latent variables. First, we processed the data following the steps suggested by SCANPY, including total count normalization, log1p logarithmization, highly variable genes extraction, a potential regression of confounding factors of genes counts, and mitochondria gene percentage, a scaling to *z*-scores, and PCA analysis. We then computed a neighborhood graph among data points and used layout ‘fa’ to generate a topologically graph. We represented the graph in diffusion map space to denoise the graph. Then Louvain clustering was performed with resolution = 1.0. PAGA was performed and the trajectory was constructed using layout ‘fa’. We chose a root cell for diffusion pseudotime and computed diffusion pseudotime using n_dcs = 10. Lastly, cells from myeloid lineages were extracted and cell density distribution along HSC/MPP-to-myeloid differentiation pseudotime was calculated and ranked from the most undifferentiated (0%) to the most differentiated (100%) states for each genotype and time point.

### Identification of genes affected by the oncogenic interactions between G12D and E2-KO

Normalized gene expression (log-transformed TPM) was calculated in each sample. For each gene, we compared its expression level in WT and G12D (or E2-KO) HSPCs to calculate the expression change in single mutant samples. Expected expression change in double mutant (G12D/E2-KO) samples was calculated as an additive change in expression level from single mutant (G12D and E2-KO) samples. Observed expression change in the double mutant (G12D/E2-KO) sample was obtained from the comparison between WT and G12D/E2-KO samples. For each gene, the expected and observed expression change was compared. In G12D/E2-KO sample, if the observed expression change of a gene matched the expected expression change based on the combined effect of each mutation alone, we concluded that the expression of the gene was not affected by oncogenic interactions between G12D and E2-KO. Conversely, if the observed expression change of a gene was significantly different from the expected expression change based on the combined effect of each mutation alone, we concluded that the expression of the gene was affected by oncogenic interactions between G12D and E2-KO. To identify genes significantly affected by the oncogenic interactions between G12D and E2-KO, we randomly shuffled the observed and expected values of gene expression changes and calculated the differences between shuffled values. We considered genes with interaction degrees below the bottom 5% or above top 5% of randomly shuffled interaction degrees as the genes significantly affected by G12D and E2-KO interactions.

### Identification of cell-type-specific genes by random forest classifier

Random forest algorithm is a machine learning method used for data classification, which works for datasets with many samples, many features, and multiple different classes^47^. For a given sample, a random forest algorithm can generate a probability for each class and the one with the highest probability would be the predicted class for the given sample. We applied a random forest algorithm in single-cell RNA-seq data to identify cell-type-specific genes by treating cells as samples, genes as features, and different cell types as different classes. We used the RandomForestClassifier package from scikit-learn v0.20.2 to build a random forest classifier and classify single cells into 11 different cell types annotated in WT HSPCs. To train a random forest classifier, feature selection was first performed by training a random forest classifier on all 15,721 expressed genes that were detected in more than three cells. We selected the 1000 most informative genes based on overall gene importance in the classifier to train the random forest classifier. We then performed 5-fold cross-validation to evaluate the performance of our trained classifier and obtained the prediction accuracy of 0.94 (Extended Data Fig. 6b). To identify HSC/MPP-specific genes in G12D/E2-KO HSPCs, we predicted the probabilities of each cell in HSC/MPP using the trained random forest classifier. We then calculated the correlation for each gene with HSC/MPP probabilities. Genes with high correlation to HSC/MPP prediction score were identified as HSC/MPP preferentially expressed genes.

### RNA-seq and ChIP experiments

For GEM knockdown, lineage-negative cells were transduced with shRNA viruses against luciferase (shLuc) or GEM (shGem). Transduced cells (GFP^+^) were FACS-sorted 72 h post-transduction and subjected to RNA-seq library preparation. RNA-seq library was prepared using the SMARTer stranded total RNA-seq pico input v2 kit (Takara). Sequencing reads were aligned to the mouse reference genome (mm10) by STAR 2.7.3.a with default parameters in the 2-pass mode^65^. Counts for each gene were generated using htseq-count v0.6.1. DEGs were identified by DESeq2 v1.14.1^66^. ChIP-qPCR experiments were performed in FACS-sorted LSK cells from WT, G12D, E2-KO, or G12D/E2-KO mouse BM^67^.

### Apoptosis and cell cycle analyses

Lineage negative cells were transduced with shRNA viruses against luciferase (shLuc) or GEM (shGem). After 72 h, cells were stained with antibodies against lineage markers (CD2, CD3, CD5, CD8, B220, Gr1, and Ter119), c-Kit, Sca-1, and Annexin V. Dead cells were excluded by DAPI positive staining. GFP^+^AnnexinV^+^DAPI^−^ LSK cells were counted as apoptotic cells. For cell cycle analysis, cells were stained with antibodies against lineage markers, c-Kit, and Sca-1, and fixed with 4% paraformaldehyde. Cells were then incubated with DAPI (1 mg/ml) before flow cytometry analysis of cell cycle in GFP^+^ LSK cells.

### BM transplantation

BM transplantation experiments were performed as previously described with modifications^11,61^. Briefly, recipient mice (CD45.1) were irradiated using an XRAD 320 X-ray irradiator with two doses of 540 rad at least 3 h apart. Cells were injected through the tail vein of anesthetized recipients. Transplanted mice were maintained on antibiotic water for 4 weeks. For transplant with GEM shRNAs with the GFP reporter, lineage negative cells from donor BM and splenic cells from moribund primary recipients were magnetically separated and transduced with two rounds of spin infection. c-Kit^+^GFP^+^ cells isolated by FACS sorting (5 × 10^5^ cells) together with supporting BM cells (CD45.1; 5 × 10^5^ cells) were injected into lethally irradiated recipient mice (CD45.1). Leukemia-initiating cell activity was measured by limiting dilution assay in NSG (NOD-*scid* L2Rg^null^) mice at 10, 100, or 1000 cells per mouse. The log-log plot and LIC frequencies were calculated using the ELDA tool^11^.

### Colony formation assays

LSK cells were isolated by FACS sorting and maintained in Prime-XV Mouse Hematopoietic Cell Medium (Irvine Scientific) with 50 µM β-Mercaptoethanol, 1% FBS, 50 ng/ml SCF, and 50 ng/ml TPO. Cells were transuded with shRNA viruses by two rounds of spin infection within 48 h. c-Kit^+^GFP^+^ cells (500 cells) were sorted and plated in methylcellulose (Cat# M3434, StemCell Technologies) with control (DMSO) or AC220 treatment (20 nM). Colonies were counted on day 7. In serial replating experiments, cells were collected and resuspended in IMDM media from plates of the previous round and seeded to the new plates as single-cell suspensions.

### Histology and immunohistochemistry (IHC)

May–Grunwald–Giemsa staining was used to analyze smear of PB or BM cells as described previously^68^. BM, spleen, and liver samples were fixed in formalin, dehydrated in gradient ethanol, and embedded in paraffin. Sectioned slides were rehydrated in gradient ethanol and visualized by standard H&E staining. For IHC analysis, antigen retrieval was performed by heating slides at 90–100 °C for 20 min in 10 mM sodium citrate buffer. Slides were cooled down to room temperature followed by washing with PBST twice. Slides were then treated with 3% H_2_O_2_ in methanol for 20 min and blocked with 5% goat serum. Slides were incubated with c-Kit (CD117) antibody (Biolegend, Cat# 105802) overnight and detected with the Elite ABC kit and DAB substrate (Vector Laboratories). Trichrome and reticulin staining was performed by the Molecular Pathology Core facility at UTSW.

### Statistical and reproducibility

Statistical details including *N*, mean, and statistical significance values are provided in the text, figure legends, or relevant methods. Error bars represent SEM or SD from either independent experiments or independent biological samples. Statistical analyses were performed using GraphPad Prism using statistical methods specified in figure legends or methods. The numbers of independent experiments or biological replicate samples and *P* values (n.s. not significant, *P < 0.05, **P < 0.01, ***P < 0.001) are provided in individual figures. *P* < 0.05 was considered statistically significant. Panels in Supplementary Figs. 1a and 3a show a representative image of at least three independent replicate samples. No statistical method was used to predetermine sample size in animal studies and the experiments were not randomized. The investigators were not blinded to allocation during experiments and outcome assessment.

### Reporting summary

Further information on experimental design is available in the Nature Research Reporting Summary linked to this article.

## Supplementary information

Supplementary Information

Description of Additional Supplementary Files

Supplementary Data 1

Supplementary Data 2

Supplementary Data 3

Supplementary Data 4

Supplementary Data 5

Supplementary Data 6

Supplementary Data 7

Reporting Summary

## Data Availability

All raw and processed scRNA-seq and bulk RNA-seq data are available in the Gene Expression Omnibus (GEO): GSE179084. Other public genomic datasets are listed in Supplementary Data 7 and deposited under accession numbers GSE112995 and GSE49847.  Source data are provided with this paper.

## Source data

Source Data

## Code availability

Raw scRNA-seq data was processed using Cell Ranger v2.0.2. Single cells were clustered using Louvain algorithm from SCANPY v1.4. KNN and UMAP visualization were performed by Spring and Seurat v3.2.0. Analysis of single-cell differentiation trajectories was conducted by PAGA from SCANPY v1.4. The custom code for random forest classification is available from GitHub (https://github.com/Yuxuan0060/Random_forest_classification). Bulk RNA-seq data analysis was conducted using STAR 2.7.3.a and DESeq2 v1.14.1. Code for analyses using other indicated software is available from the websites of the corresponding software.
