# Supplementary material for: Convergence of oncogenic cooperation at single-cell and single-gene levels drives leukemic transformation
Source: Nat Commun. 2021 Nov 3;12:6323. doi: 10.1038/s41467-021-26582-4 (PMC8566485; doi:10.1038/s41467-021-26582-4)
Supplement: Supplementary file 1 — Supplementary Information [file 41467_2021_26582_MOESM1_ESM.pdf]

Supplementary Information

Convergence of Oncogenic Cooperation at Single-Cell and Single-Gene Levels Drives Leukemic Transformation

Liu and Gu et al.

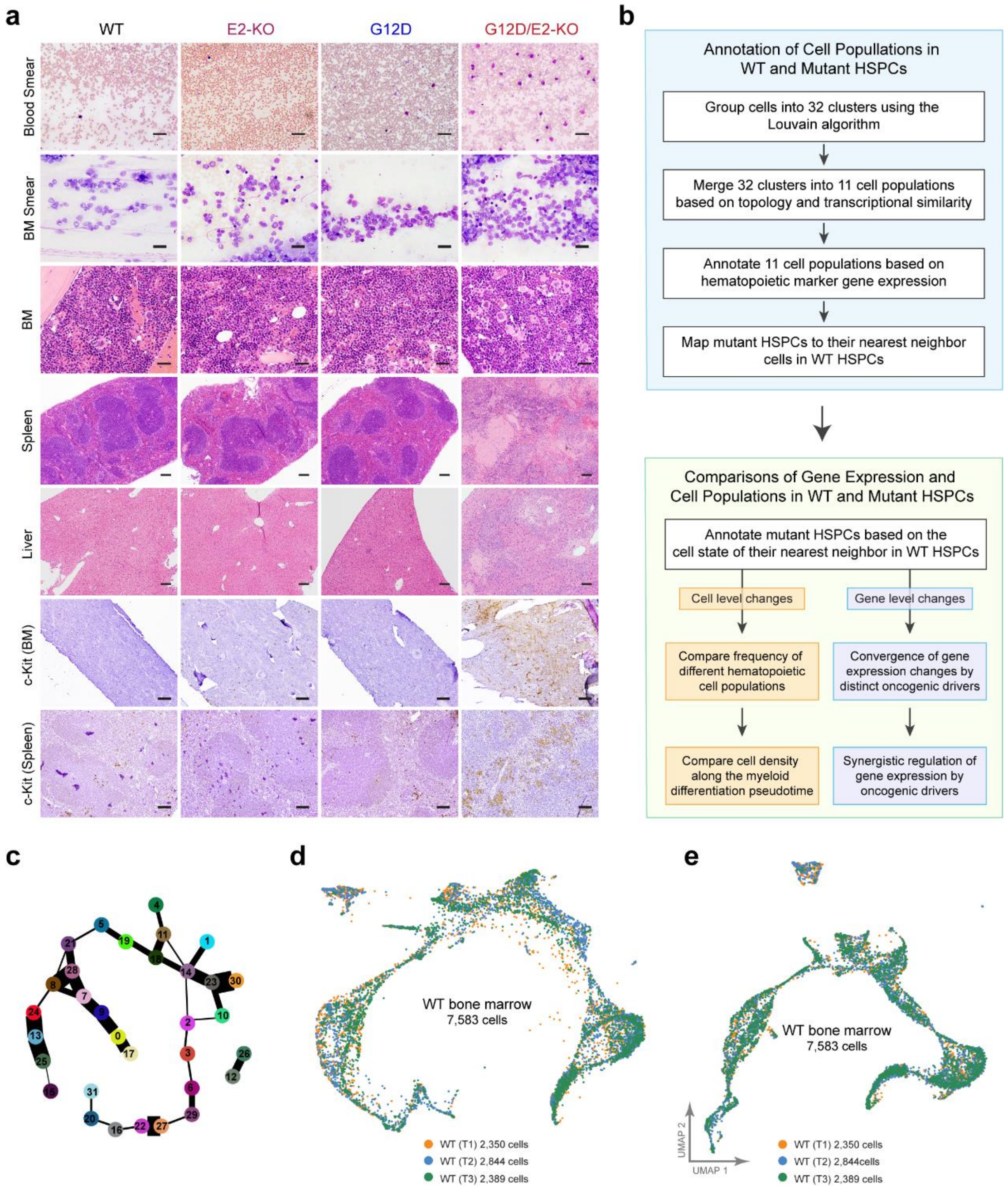

Supplementary Figure 1 | An integrative approach to capture the kinetics of single-cell transcriptomes during leukemia progression.

a, Histopathological analysis of mice with the indicated genotypes. Representative images of peripheral blood and bone marrow smear 10 weeks post-pIpC or when the mice were moribund (scale bars, 20 μm). Representative images are shown for H&E staining of BM, spleen and liver (scale bars, BM 25 μm, spleen and liver 150 μm) and immunochemistry staining of c-Kit in BM and spleen (scale bars, 20 μm) 10 weeks post-pIpC or when the mice were moribund.
b, The workflow of scRNA-seq data processing and analysis, including the annotation of WT and HSPCs and the comparisons of cell and gene level changes between WT and mutant HSPCs.
c, PAGA visualization of WT HSPCs. Cells are grouped and colored by Louvain cluster in Scanpy.
d, KNN visualization of WT HSPCs at different time points. Each dot represents a single cell color-coded by different time points (T1, T2 or T3).
e, UMAP visualization of WT HSPCs at different time points.

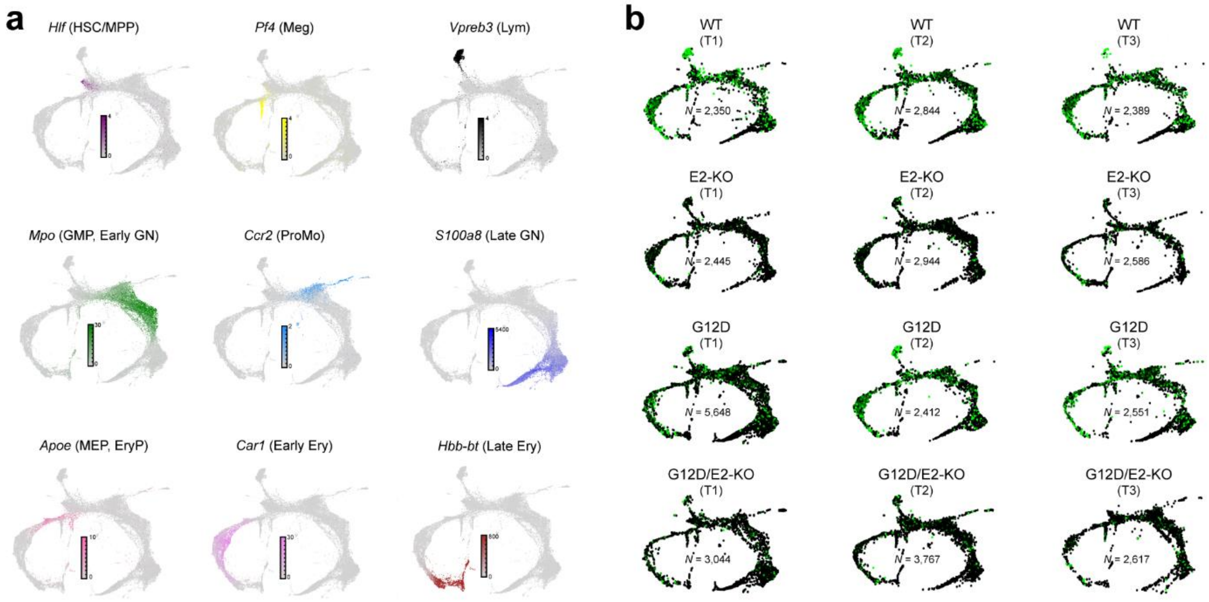

Supplementary Figure 2 | Expression of lineage marker genes and EZH2 on the differentiation trajectories.
a, KNN visualization of lineage-specific gene expression in single cells from all 12 samples. Each dot represents a single cell. Color scales show the expression levels of the indicated genes.
b, KNN visualization of *Ezh2* mRNA expression in single cells from samples of different genotypes at different time points. Each dot represents a single cell. Color scales show the expression levels of *Ezh2*.

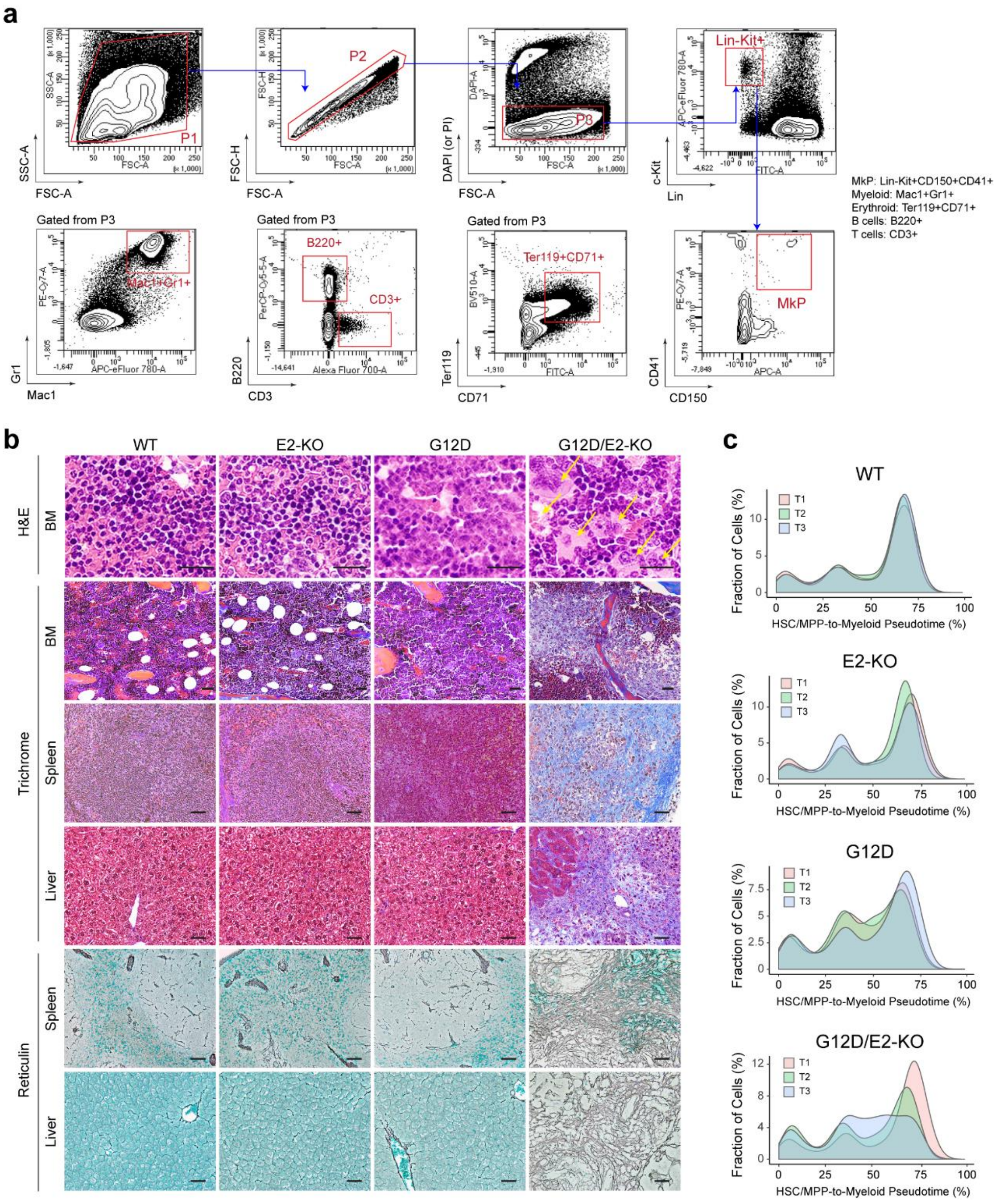

Supplementary Figure 3 | Development of primary myelofibrosis in G12D/E2-KO mice.

a, Representative flow cytometry gates are shown for the analysis of various hematopoietic lineages in mouse bone marrow.

b, Representative images are shown for H&E (*top*, scale bars, 25 µm) and Trichrome staining (*bottom*, scale bars, 10 µm) of mouse bone marrows at 10 weeks post-pIpC or moribund. Arrowheads indicate the hypolobulated dysplastic megakaryocytes. Trichrome and reticulin staining of spleen and liver sections are also shown for the indicated genotypes 10 weeks post-pIpC or moribund (scale bars, 150 µm).

c, Cell density distribution along the HSC/MPP-to-myeloid differentiation pseudotime for each genotype (WT, E2-KO, G12D, and G12D/E2-KO) at different time points (T1 to T3).

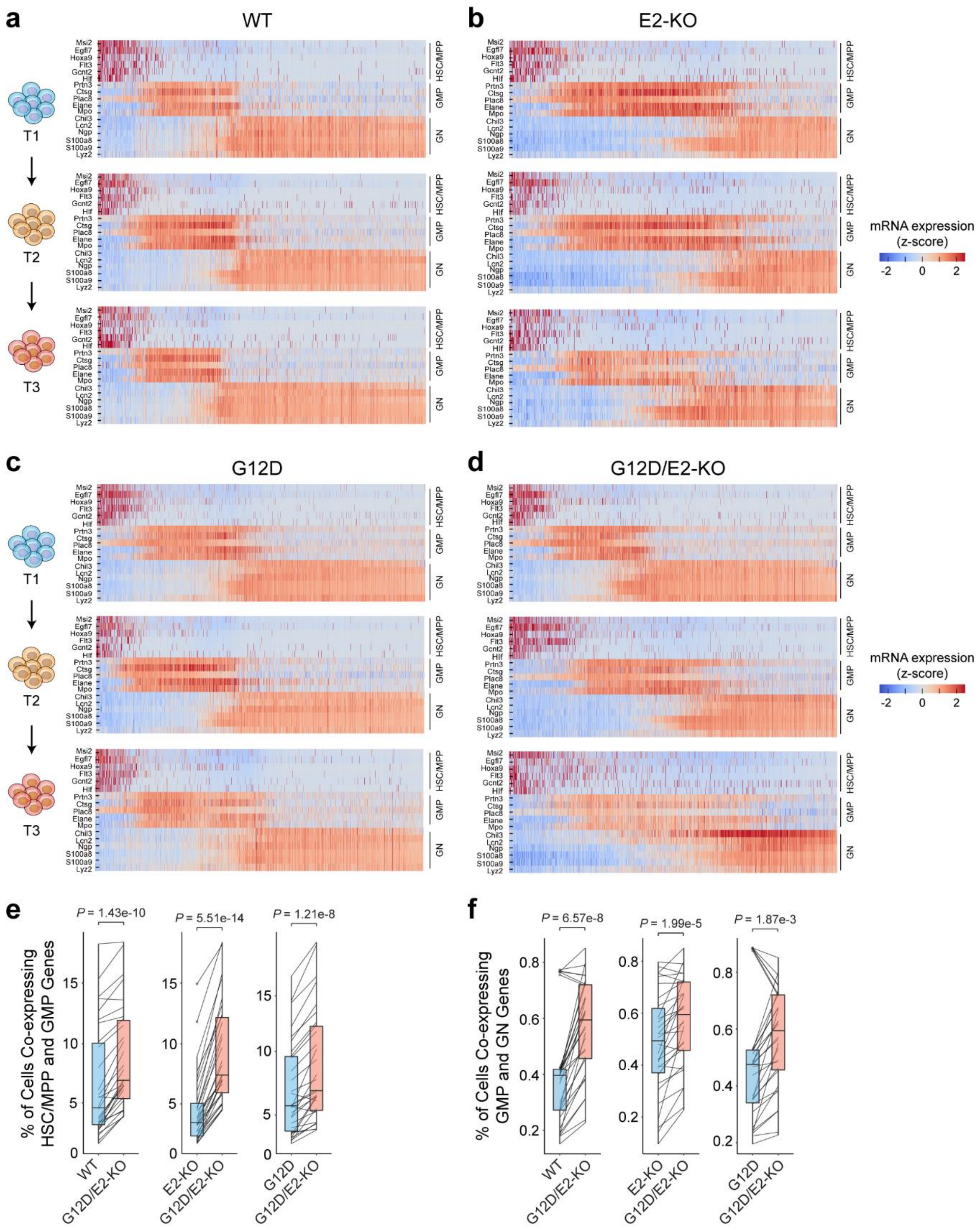

Supplementary Figure 4 | Co-expression of stemness and myeloid-priming genes at single-cell level in G12D/E2-KO HSPCs.

a, Expression of marker genes for HSC/MPP, GMP and granulocytes (GN) along the HSC/MPP-to-myeloid differentiation pseudotime in WT HSPCs at different time points (T1 to T3). Heatmap shows the normalized mRNA expression levels.

b, Expression of marker genes for HSC/MPP, GMP and GN along the HSC/MPP-to-myeloid differentiation pseudotime in E2-KO HSPCs at different time points (T1 to T3).
c, Expression of marker genes for HSC/MPP, GMP and GN along the HSC/MPP-to-myeloid differentiation pseudotime in G12D HSPCs at different time points (T1 to T3).
d, Expression of marker genes for HSC/MPP, GMP and GN along the HSC/MPP-to-myeloid differentiation pseudotime in G12D/E2-KO HSPCs at different time points (T1 to T3).
e, Analysis of the frequencies of cells co-expressing HSC/MPP and GMP genes in WT, E-KO, G12D, and G12D/E2-KO samples. Each dot represents the frequency of cells co-expression one pair of HSC and GMP genes. Boxes show median of the data and quartiles, and whiskers extend up to 1.5x of the interquartile range. *P* values were calculated by a two-sided paired *t* test.
f, Analysis of the frequencies of cells co-expressing GMP and granulocyte (GN) genes in WT, G12D, E2-KO, and G12D/E2-KO samples. Boxes show median of the data and quartiles, and whiskers extend up to 1.5x of the interquartile range. *P* values were calculated by a two-sided paired *t* test.

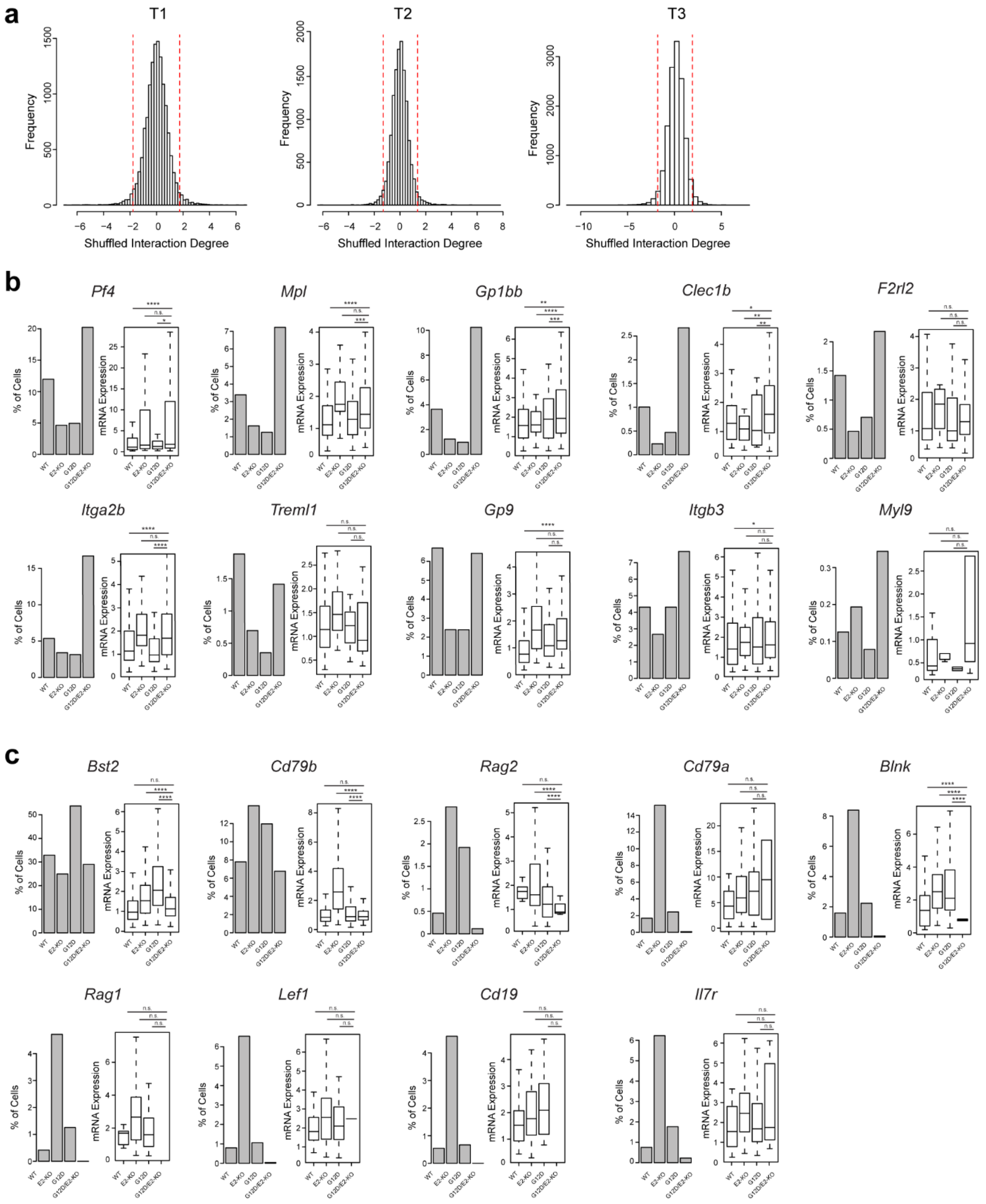

Supplementary Figure 5 | EZH2 and NRAS mutations cooperate to regulate the expression of platelet and B cell genes.

a, Histogram of permuted interaction scores is shown. The observed and expected expression changes for all genes were randomly shuffled, and the differences between all permuted pairs were calculated as the permuted interaction scores. *Red* lines indicate the top and bottom 5% cutoff of the permuted interaction scores.

b, EZH2 and NRAS mutations cooperate to regulate expression of platelet genes. For each gene, the fraction (%) of cells with gene expression greater than 0 (left) and the mRNA level (right) are shown in WT, E2-KO, G12D, and G12D/E2-KO HSPCs, respectively. Boxes show median of the data and quartiles, and whiskers extend up to 1.5x of the interquartile range. Sample numbers with *Pf4* expression level greater than 0 are 286, 120, 126 and 529 in WT, E2-KO, G12D and G12D/E2-KO, respectively. Sample numbers for *Mpl* are 81, 42, 32 and 189, for *Gp1bb* 87, 32, 25 and 268, for *Clec1b* 24, 6, 12 and 70, for *F2rl2* 34,12, 18 and 57, for *Itga2b* 128, 88, 80 and 457, for *Treml1* 47, 18, 9 and 37, for *Gp9* 160, 62, 61 and 168, for *Itgb3* 103, 69, 110, and 201, and for *Myl9* 3, 5, 2 and 9 in WT, E2-KO, G12D and G12D/E2-KO, respectively. P values were calculated by two-sided t test. *$P < 0.05$; **$P < 0.01$; ***$P < 0.001$; ****$P < 0.0001$; n.s. not significant.

c, EZH2 and NRAS mutations cooperate to regulate expression of B cell genes. Sample numbers with *Bst2* expression level greater than 0 is 786, 645, 1371 and 759 in WT, E2-KO, G12D and G12D/E2-KO, respectively. Sample numbers for *Cd79b* are 186, 361, 305 and 177, for *Rag2* 11, 73, 49 and 3, for *Cd79a* 40, 393, 62 and 2, for *Blnk* 38, 217, 57 and 2, for *Rag1* 10, 32, 122 and 0, for *Lef1* 19, 169, 27 and 1, for *Cd19* 13,119,17 and 0, and for *Il7r* 18, 161, 45, and 6 in WT, E2-KO, G12D and G12D/E2-KO, respectively.

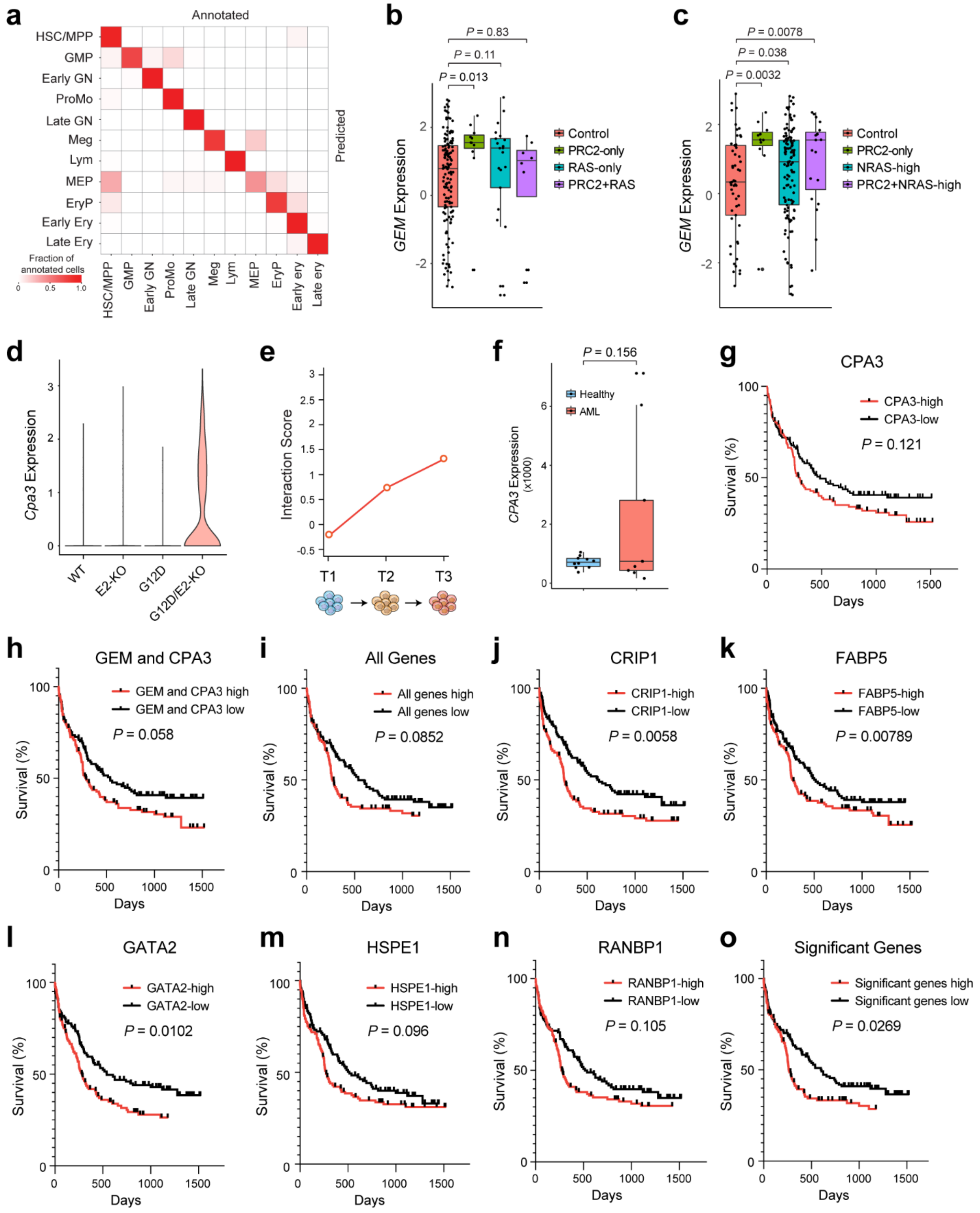

Supplementary Figure 6 | Identification of candidate regulators of leukemia-initiating cells.

a, Five-fold cross-validation of the random forest classifier. Color scale indicates the fraction of annotated cells. Cells falling on and off the diagonal are correctly classified (94%) and mis-labelled (6%), respectively.

b, Correlation of *GEM* expression (log2 RPKM) with PRC2 and/or RAS mutations in AML. AML samples without PRC2 and RAS mutations (control), PRC2 mutations alone (PRC2-only), RAS mutations alone (RAS-only), and both PRC2 and RAS mutations (PRC2+RAS) were compared. PRC2-only and RAS-only samples with mutant allele frequencies higher than 0.25 were used.

c, Correlation of *GEM* expression (log2 RPKM) with PRC2 mutations and/or NRAS expression in AML. AML samples without PRC2 and lower NRAS expression (bottom 50%, control), PRC2 mutations alone (PRC2-only), NRAS-high expression (top 50%, NRAS-high), and both PRC2 mutations and NRAS-high expression (PRC2+NRAS-high) were compared. PRC2-only samples with mutant allele frequencies higher than 0.25 were used.

d, Violin plot of *Cpa3* mRNA expression in HSC/MPP of different genotypes.

e, The interaction scores of G12D and E2-KO on *Cpa3* expression.

f, Expression of *CPA3* is upregulated in AML samples relative to HSPCs from healthy donors.

g, Increased *CPA3* associates with poor survival in the AML cohort (GSE12417). Patients were ranked by *CPA3* mRNA and divided to CPA3-low (bottom 50%) and CPA3-high (top 50%) groups.

h, Increased *GEM* and *CPA3* associates with poor survival in AML. Patients were ranked by the summation of *GEM* and *CPA3* mRNA levels and divided to bottom and top 50% groups.

i, Increased expression of all 19 of 32 LIC-related genes associates with poor survival in AML.

j-n, Increased expression of *CRIP1*, *FABP5*, *GATA2*, *HSPE1*, and *RANBP1* associates with poor survival in AML.

o, Increased expression of all significant genes (*GEM*, *CPA3*, *CRIP1*, *FABP5*, *GATA2*, *HSPE1*, and *RANBP1*) associates with poor survival in AML.

For **b**, **c**, and **f**, Boxes show median of the data and quartiles, and whiskers extend to 1.5x of the interquartile range. *P* values were calculated by a two-sided *t* test. For **g-o**, *P* values were calculated for all quantiles using log-rank (Mantel-Cox).

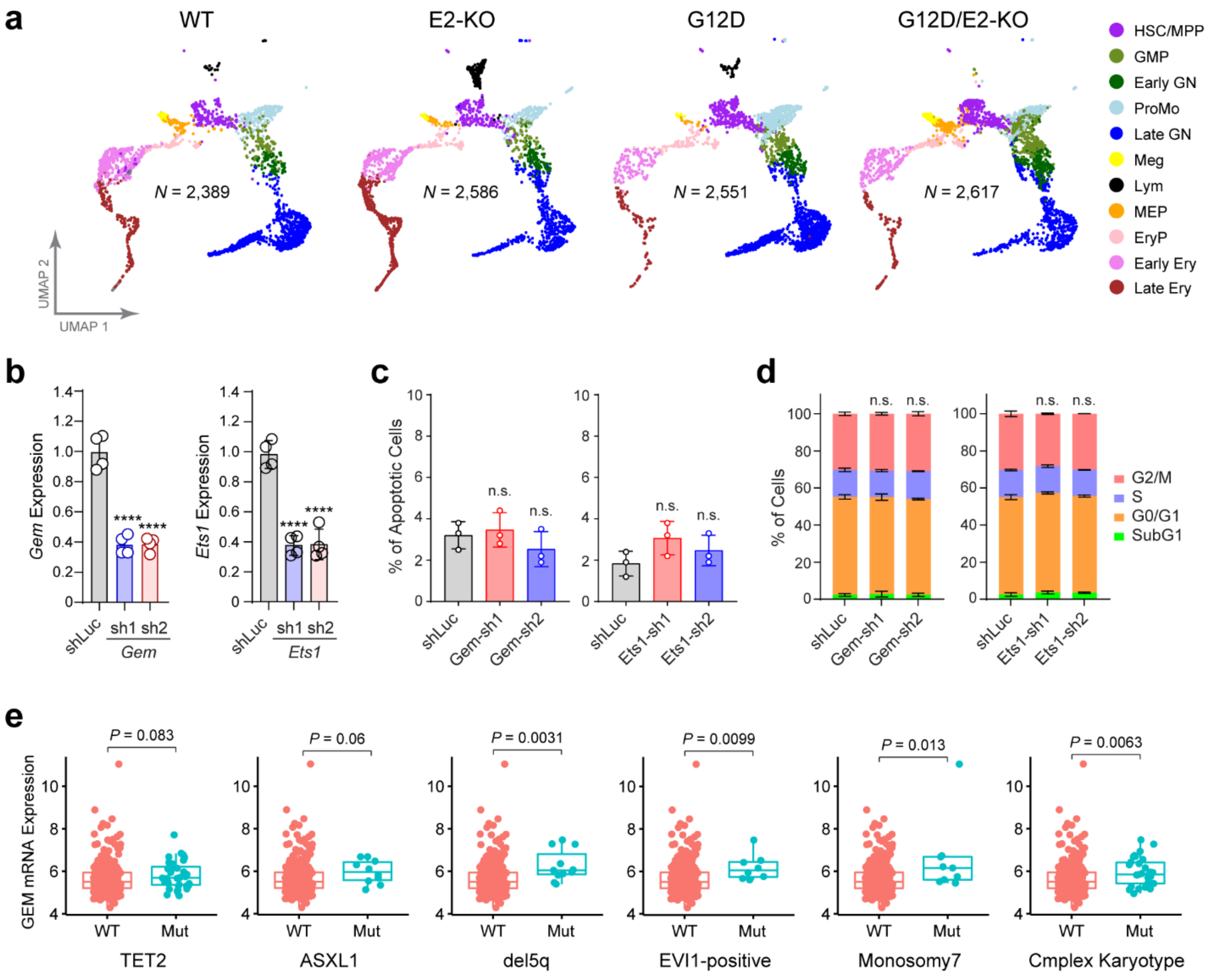

Supplementary Figure 7 | Dysregulation of GEM expression in myeloid malignancies.

a, UMAP visualization of single-cell transcriptomes from HSPCs of the indicated genotypes at time point T3. Each dot represents a single cell colored by the cell-type annotations.

b, Depletion of *Gem* or *Ets1* in WT HSPCs by independent shRNAs (sh1 and sh2). Results are mean ± SD and analyzed by one-way ANOVA. *****P* < 0.0001.

c, GEM or ETS1 depletion had no effect on apoptosis (Annexin V$^+$) in WT LSK cells. Results are mean ± SD (*N* = 3 independent experiments) and analyzed by one-way ANOVA with Dunnett's test. n.s. not significant.

d, GEM or ETS1 depletion had no effect on cell cycle in WT LSK cells. Results are mean ± SD (*N* = 3 independent experiments) and analyzed by two-way ANOVA with Dunnett's test. n.s. not significant.

e, Expression of *GEM* is shown in AML subtypes associated with different genetic lesions and/or cytogenetic risk groups from the ECOG 1900 AML cohorts. Wilcoxon *P* values were calculated for comparisons between wild-type (WT) and mutant (Mut) AML samples with the indicated lesions. Source data are provided as a Source Data file.
