# Supplementary material for: Convergence of oncogenic cooperation at single-cell and single-gene levels drives leukemic transformation
Source: Nat Commun. 2021 Nov 3;12:6323. doi: 10.1038/s41467-021-26582-4 (PMC8566485; doi:10.1038/s41467-021-26582-4)
Supplement: Supplementary file 2 — Description of Additional Supplementary Files [file 41467_2021_26582_MOESM2_ESM.pdf]

File Name: **Supplementary Data 1**
Description: **Quality control analysis of scRNA-seq datasets.** The numbers of cells, detected genes, mean reads per cell, median genes per cell, and mean UMI counts per cell are shown.

File Name: **Supplementary Data 2**
Description: **Annotation of cell populations by the expression of established hematopoietic marker genes.** The lists of cell populations and corresponding marker genes are shown.

File Name: **Supplementary Data 3**
Description: **List of differentially expressed genes between and within states.** The numbers of differentially expressed genes (DEGs) between and within cell states are shown.

File Name: **Supplementary Data 4**
Description: **List of differentially expressed genes upon GEM depletion.** The lists of differentially expressed genes (DEGs) between control (shLuc) and GEM depletion (shGem) in WT or G12D/E2-KO HSPCs are shown.

File Name: **Supplementary Data 5**
Description: **Sequences of primers and shRNAs.** The name and sequence of each primer or shRNA are shown.

File Name: **Supplementary Data 6**
Description: **List of key reagents and resources.** The lists of antibodies, reagents, recombinant DNA and resources are shown.

File Name: **Supplementary Data 7**
Description: **List of genomic datasets used in this study.** The name, data type, cell type, GEO accession number and citation are shown.
